# Evolution of the T-Cell Receptor (TR) Loci in the Adaptive Immune Response: The Tale of the TRG Locus in Mammals

**DOI:** 10.3390/genes11060624

**Published:** 2020-06-05

**Authors:** Rachele Antonacci, Serafina Massari, Giovanna Linguiti, Anna Caputi Jambrenghi, Francesco Giannico, Marie-Paule Lefranc, Salvatrice Ciccarese

**Affiliations:** 1 Department of Biology, University of Bari “Aldo Moro”, 70124 Bari, Italy; giovanna.linguiti@uniba.it (G.L.); salvatricemaria.ciccarese@uniba.it (S.C.); 2 Department of Biological and Environmental Science and Technologies, University of Salento, 73100 Lecce, Italy; sara.massari@unisalento.it; 3 Department of Agricultural and Environmental Science, University of Bari “Aldo Moro”, 70124 Bari, Italy; anna.caputijambrenghi@uniba.it (A.C.J.); francesco.giannico@uniba.it (F.G.); 4 IMGT^®^, the International ImMunoGeneTics Information System^®^, Laboratoire d’ImmunoGénétique Moléculaire LIGM, Institut de Génétique Humaine IGH, UMR9002 CNRS, Université de Montpellier, CEDEX 5, 34396 Montpellier, France; marie-paule.lefranc@igh.cnrs.fr

**Keywords:** T cell receptor, TRG locus, TRG genes, immunogenomics, evolution, mammals, IMGT

## Abstract

T lymphocytes are the principal actors of vertebrates’ cell-mediated immunity. Like B cells, they can recognize an unlimited number of foreign molecules through their antigen-specific heterodimer receptors (TRs), which consist of αβ or γδ chains. The diversity of the TRs is mainly due to the unique organization of the genes encoding the α, β, γ, and δ chains. For each chain, multi-gene families are arranged in a TR locus, and their expression is guaranteed by the somatic recombination process. A great plasticity of the gene organization within the TR loci exists among species. Marked structural differences affect the TR γ (TRG) locus. The recent sequencing of multiple whole genome provides an opportunity to examine the TR gene repertoire in a systematic and consistent fashion. In this review, we report the most recent findings on the genomic organization of TRG loci in mammalian species in order to show differences and similarities. The comparison revealed remarkable diversification of both the genomic organization and gene repertoire across species, but also unexpected evolutionary conservation, which highlights the important role of the T cells in the immune response.

## 1. Introduction

T lymphocytes play a crucial role in the immune surveillance of all jawed vertebrates. There are essentially two major subpopulations of T cells, which are classified as or according to the T-cell receptors (TRs) expressed on their membrane [1]. Each T lymphocyte expresses a unique antigen-specific TR.

αβ T cells are the most abundant in the explored species, with a broad distribution in circulation and lymphoid organs. αβ T cells are mainly able to recognize peptides from degraded proteins bound to major histocompatibility (MH) proteins at the surface of antigen-presenting cells. In this context, αβ T cells comprise two subsets based on the expression of CD4 or CD8 coreceptors, which participate in the recognition of the MH-peptide antigen and in the T-cell signal transduction. CD4+ T cells recognize ligands in the context of MH class II (MH2) proteins, whereas CD8+ T cells recognize antigens via presentation by MH class I (MH1) proteins [2].

γδ T cells are an enigmatic cell population with unique features compared with αβ T cells [3]. The amount of circulating γδ T cells varies in different species. In humans and mice (“γδ-low species”), γδ T cells make up only 1–10% of circulating T lymphocytes, although they are substantially enriched in mucosa and epithelial tissues, suggesting a critical role in early immune responses. In contrast, in “γδ-high species” such as chickens, cattle, sheep, and pigs, γδ T cells constitute a major lymphocyte population in the peripheral blood (15–60%), particularly in young animals [4,5,6,7,8], suggesting an important role in host defense. γδ T cells are a functionally heterogeneous population, although details of their functions are becoming clearer. They recognize antigens directly and without processing, in a manner similar to immunoglobulins (IG) [9], or presented by RPI-MH1Like proteins [10], providing more flexible and not yet clear recognition of a wide range of ligands (soluble, membrane-bound, and unprocessed antigens)**.** In line with their MH-independent antigen recognition, γδ T cells lack CD4 and CD8, and are therefore referred to as CD4 and CD8 double-negative cells. However, in ruminants, a large proportion of circulating γδ T cells express the γδ-T-cell specific WC1 proteins, which belong to the scavenger superfamily and act as pattern-recognition receptor (PRRs) and γδ-T-cell-activating coreceptors [11,12]. 

In all species, γδ T cells are involved in diverse and important roles in not only adaptive, but also innate immune responses, specific to each anatomical location and physiological context. Defined roles for γδ T cells are few. For example, they have been shown to play a significant function in wound healing in mouse epidermis [13]. A restricted subset of γδ T cells in human peripheral blood are involved in the recognition of phosphoantigen metabolites (PAgs), which can be produced by bacterial and eukaryotic cells as result of metabolic dysregulation [14,15,16]. In general, the ability of γδ T cells to recognize cell-surface molecules expressed on stressed or infected cells has direct consequences for the function of these cells in a broad range of immune settings, including antimicrobial immunity, anti-tumor immunity, and tissue homeostasis [17,18]. In ruminants, the γδ T cells recognize infectious agents in combination with WC1 proteins, making these species a valuable model for understanding the variety of ways in which these T cells respond to important pathogens [19].

The functional differences of γδ T cells among species mirror differences in the TR γ (TRG) and TR δ (TRD) chain repertoires, with a wider and more diversified repertoire in domestic species compared to humans and mice. In turn, the TRG and TRD chain repertoires depend on the gene organization of the TRG and TRD loci which, interestingly, show more variability between the different species than do the TRA and TRB loci, which encode the and chains of the T cells. The MH-independent recognition of T cells with respect to the MH restriction of T cells probably explains the plasticity of the TR and TR genomic organization.

## 2. The TR Chains are Encoded by Separate Multigene Families

αβ and γδ heterodimers are members of the Ig superfamily, and the genes encoding for each TR chain [1] are organized in the germline DNA in a manner remarkably similar to the multigene organization of the immunoglobulin (IG) loci [2,9]. Each TR locus lies on a different chromosome region and consists of arrays of different gene types, including variable (*V*), diversity (*D*), joining (*J*), and constant (*C*) genes, each representing a multigene sub-family. Productive TRs are produced during the development of T lymphocytes in the thymus by somatic rearrangements of *V* and *J* genes in the TRA and TRG loci, and between *V*, *D*, and *J* genes in the TRB and TRD loci. After transcription, the resulting rearranged V-(D)-J region, encoding the variable domain of the TR chain, is spliced to the *C* gene, which encodes the constant domain of the receptor. The variable domain forms the antigen-binding site, while the constant domain anchors the receptor to the cell membrane and is involved in signal transduction. The resulting chain is a protein with the variable domain composed of seven distinguishable regions: three hypervariable loops or complementarity-determining regions (CDR) and four framework regions (FR). Two of the CDR loops, CDR1 and CDR2, are encoded by the *V* gene. The third CDR loop (CDR3) reflects the ability of the *V* gene to rearrange to any (*D*) *J* gene [1]. Therefore, the number of *V*, *D*, and *J* genes in the germline DNA, and the somatic V-(D)-J rearrangement mechanism unique to the adaptive immune response, contribute to the huge diversity of the expressed TR repertoire, allowing potentially billions of different TR antigen-binding sites to be produced from a limited set of genes [2].

The gene organization in each of the four TR loci is known for many species. The genomic structure of the TRB locus has a common feature in representative species of several orders of eutherian mammals, with a pool of *V* (*TRBV*) genes, a different number from species to species, positioned upstream of tandem-aligned TRBD-J-C clusters, each composed of a single *D* (*TRBD*) gene, several *J* (*TRBJ*) genes, and one *C* (*TRBC*) gene. A single *TRBV* gene in inverted orientation of transcription completes each TRB locus at the 3′ end. In most mammalian species, including human [20,21], mouse [22], chimpanzee and rhesus monkey [23], dog [24], rabbit [25], ferret [26], and cat [27], two TRBD-J-C clusters exist. In contrast, in the cetartiodactyl lineage [28,29,30,31,32,33,34,35], a duplication event within the 3′ end of the TRB locus led to the generation of a third TRBD-J-C cluster, increasing the number of *TRBD* and *TRBJ* genes available for the somatic rearrangements. 

The organization of the *TRA* and *TRD* genes displays an intriguing and conserved feature, in that they are located at a single chromosomal region with the TRD locus nested within the TRA locus. In fact, this is referred to the TRA/TRD locus. Despite this complex arrangement, each TR locus exhibits specific control of its own gene assembly. The general genomic organization of the TRA/TRD region, from the 5′ end to the 3′ end, consists of an array of *TRAV* genes among which the *TRDV* genes are embedded. The region continues with the TRD locus, i.e., the *TRDD*, *TRDJ*, and one *TRDC* gene, followed by a *TRDV* gene in inverted orientation of transcription. At the 3′ end, a cluster of *TRAJ* lies followed by one *TRAC*, which completes the TRA locus. As a consequence of the gene organization, the whole TRD locus is excised from the genomic sequence when the first TRA V-J rearrangement occurs in the TRA/TRD locus. The characterization of the TRA/TRD locus in the different mammals (https://www.imgt.org/IMGTrepertoire/: *Homo sapiens*, *Mus musculus*, *Macaca mulatta*, *Bos taurus*; [27,36,37,38,39,40]), has shown that in this case, the number of *TRAV* and *TRDV* genes also represents the major disparity among species.

Differently from the TRB and TRA/TRD loci, the TRG locus shows great gene-organization plasticity related to the evolution of different species. Typically, the TRG genes comprise an array of multiple *TRGV* genes linked to *TRGJ* and *TRGC* genes organized in J-C clusters; otherwise, the TRG locus features a gene-cluster organization in V-J-C rearrangement units or cassettes. 

The main goal of this review was to collect all the data on the TRG locus structure of eutherian mammalian species for which the genomic organization has been characterized in detail, and to highlight differences and similarities. The *Homo sapiens* TRG locus is described as a paradigm, as it was the first complete locus of the adaptive immune response to be entered in databases as “genes” as well as conventional genes, leading in 1989 to the creation of IMGT and to immunoinformatics, a new science at the interface between immunogenetics and bioinformatics [2]. On the basis of the accepted phylogeny, we describe the genomic characteristic of the TRG locus in Cetartiodactyla/Carnivora lineages, with respect to Rodentia/Lagomorpha/Primata. Furthermore, for a comparative analysis, the genomic organization of the TRG locus in other vertebrate species, outgroups respective to placental mammals, is also described.

## 3. The *Homo sapiens* TRG Locus

**Figure 1:**
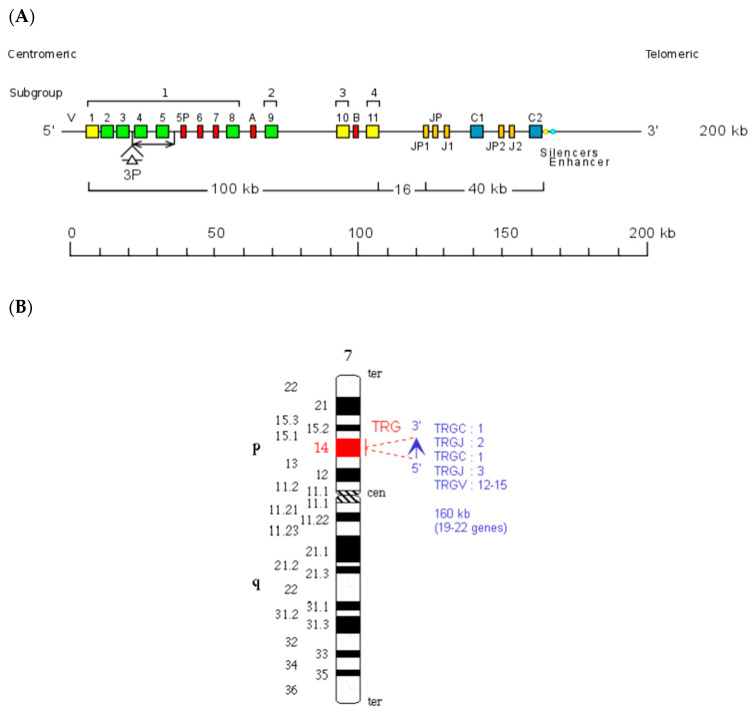
Human (*Homo sapiens*) TRG locus representation (**A**) and chromosomal localization (**B**), reproduced with permission from IMGT^®^ (http://www.imgt.org). In (**A**), colors are according to IMGT color menu for genes (https://www.imgt.org/IMGTScientificChart/RepresentationRules/colormenu.php#LOCUS). The boxes representing the genes are not to scale. Exons are not shown. A double arrow indicates insertion/deletion polymorphisms. The *Amphiphysin* (*AMPH*) (IMGT 5′ borne) was identified 16 kb upstream of *TRGV1* (ORF), the most 5′ gene in the locus, and the *Related to steroidogenic acute regulatory protein D3-N-terminal like* (*STARD3NL*) (3′ IMGT borne) was identified 9.4 kb downstream of *TRGC2* (F), the most 3′ gene in the locus. In (**B**), a vertical red line indicates the localization of the TRG locus at 7p14. A blue arrow indicates the orientation 5′ → 3′ of the locus, and the gene group order in the locus. The blue arrow is proportional to the size of the locus, indicated in kilobases (kb). The total number of genes in the locus is shown in parentheses.

The *Homo sapiens* TRG locus at 7p14 spans 160 kb [1,41] (Figure 1A, with permission of IMGT^®^ http://www.imgt.org). The orientation of the human TRG locus on the chromosome is reversed (REV) (Figure 1B, with permission of IMGT^®^ http://www.imgt.org). It consists of 12–15 *TRGV* genes upstream of a duplicated J-C cluster, which comprises in the first part three *TRGJ* genes and the *TRGC1* gene, and in the second part, two *TRGJ* genes and the *TRGC2* gene [42,43,44,45,46,47,48,49,50,51]. The 5′-most *TRGV* genes occupy the most centromeric position, whereas the *TRBC2* gene, 3′ of the locus, is the most telomeric in the TRG locus. The *TRGV* genes belong to six different subgroups based on the absence of cross-hybridization between them and defined, in terms of sequences, by less than 75% identity at the nucleotide level in their V regions [44]. *TRGV9*, expressed in 80–95% of the human peripheral T cells, is the unique member of Subgroup 2. *TRGV10* and *TRGV11*, the single members of subgroups 3 and 4, respectively, have been found to be rearranged and transcribed, but they are open reading frames (ORFs) that cannot be expressed in a gamma chain, due to a splicing defect of the pre-messenger [52,53]. The potential repertoire consists of four to six functional *TRGV* genes belonging to two subgroups, five *TRGJ* and two *TRGC* genes [54,55]. The description of the germline V region and that of the expressed rearranged V-J domain and CDR lengths is based on the IMGT unique numbering (CDR-IMGT) for the V domain [56,57,58] and IMGT Collier de Perles [59], whereas that of the C-γ domain is based on the IMGT unique numbering for the C domain [60].

Polymorphisms in the number of *TRGV* genes and in the exon number of the *TRGC2* gene have been described in different populations. Variation of the number of TRGV subgroup genes (from 7 to 10) has been observed [61,62]. These allele polymorphisms, which result from the deletion of *V4* and *V5*, or from the insertion of an additional gene, *V3P*, between *V3* and *V4*, can be detected via restriction-fragment length polymorphism (RFLP). The two *TRGC* genes, which are 16 kb apart, resulted, with their associated *TRGJ* genes, from a recent duplication in the locus. However, there are structural differences. *TRGJP1*, *TRGJ1*, and *TRGC1* cross-hybridize to *TRGJP2*, *TRGJ*, and *TRGC2*, respectively, whereas the *TRGJP* has no equivalent in the TRGJP2-J2-C2 cluster. The *TRGC* genes encode the constant domain (C-γ) of 110 amino acids (AA), the connecting region (CO), the transmembrane region (TM), and the cytoplasmic region (CY). The *TRGC1* has three exons and encodes a C-region of 173 AA, whereas the *TRGC2* gene has four or five exons, owing to the duplication or triplication of a region that includes Exon 2 (EX2, EX2T and/or EX2R) and encodes a C-region of 189 or 205 AA, respectively [1]. This allelic polymorphism of the *TRGC2* with duplication (C2(2×)) or triplication (C2(3×)) of Exon 2 can be identified via RFLP [63]. Exon 2 of the *TRGC1* gene has a cysteine involved in the interchain disulfide bridge, whereas the cysteine is not conserved in Exon 2 of the human *TRGC2* gene. Enhancer and silencer sequences have been characterized 6.5 kb downstream of the *TRGC2* gene [64]. The total number of *TRG* genes per haploid genome in human is 19 to 22, of which 11 to 13 are functional.

## 4. The “Gene Cluster” Organization of the TRG Locus Is Predominant in the Outgroups

The first paper on the chicken (*Gallus gallus*), reported the screening of a splenic cDNA library and Northern blot analysis of the thymus and spleen. Results identified three multimember TRGV subgroups, three *TRGJ* genes, and a single constant *TRGC* gene in the TRG locus [65]. At that time, the genomic organization of the TRG loci was known only for mice [66,67,68] and humans [41,42,45], and a TRGV gene expansion was proposed to explain the association between the high frequency of γδ T cells in chickens as well as in cattle, sheep, and pigs [4,5,6,7], respective to the low frequency of γδ T cells in mice and humans. In a very recent paper, the *Gallus gallus* TR genomic organization was obtained by using Illumina and single-molecule real-time sequencing technology to re-sequence genomic regions of chicken TR loci based on 10 mapped bacterial artificial chromosome clones. The chicken TRG locus has been mapped to Chromosome 2 and it spans only 82 kb; *DNA-dependent protein kinase catalytic subunit* (*PRKDC*) and *leucine-rich repeat flightless-interacting protein 2* (*LRRFIP2*) genes are found immediately flanking the 5′ and 3′ end of the TRG locus (Table 1, Supplementary Figure S1A) [69]. The chicken TRG locus organization recalls the cluster scheme. It consists of a single J-C (three *TRGJ*–one *TRGC*) gene cluster equipped with 37 upstream *TRGV* genes that are divided into 11 subgroups (Table 1, Supplementary Figure S1A). This expansion of *TRGV* genes is slightly at odds with their usage in the peripheral blood, where only 15 have been found to be expressed [69]. The single *TRGC* gene is made up of three exons.

Recently, the genomic organization of the TRG locus and the germline and expressed repertoire of *TRG* genes in the White Peking duck were determined. In this avian, the TRG locus consists of 13 *TRGV* genes classified into six subgroups upstream of a single J-C (five *TRGJ*–one *TRGC*) gene cluster (Table 1, Supplementary Figure S1B) [70]. The total number of variables is certainly lower than that found in chickens, although this number may not be the correct one. In fact, the 5′ end of the locus lacks the *PRKDC* flanking gene, and this is an indication of the genomic incompleteness of the locus itself.

In the sandbar shark (*Carcharhinus plumbeus*), a cartilaginous fish, the TRG locus consists of a single J-C (three *TRGJ*–one *TRGC*) gene cluster preceded by five *TRGV* genes (Table 1, Supplementary Figure S1C) [71]. Approximately equal numbers of clones have been found containing *TRGV1* (18), *TRGV2* (19), *TRGV3* (12), and *TRGV4* (17), indicating that there is no bias in the rearrangement of these *V* genes; however, the author found only four clones expressing *TRGV5*, suggesting that there may be significantly less rearrangement of this most 5′ distal *V* gene. Similarly, no bias was apparent in the expression of the three *TRGJ* genes. However, expression analysis conducted on spleen tissue from a single shark revealed the presence of a high degree of nucleotide mutations in the V region of cDNA sequences with respect to parental genomic sequences. Somatic hypermutation (SHM) in the sandbar shark TRG V region is the explanation for these data. The SHM process has shark-specific characteristics, such as the presence of tandem mutations [72].

**Table 1:** Genomic organization and gene content of the TRG locus in the outgroups.

		TRGV Genes	TRGJ Genes	TRGC Genes	Chromosomal Localization	Miniature Locus	References
**BIRDS**	Chicken	TRGV1 (6) TRGV2 (7) TRGV3 (2) TRGV4 (5) TRGV5 (2) TRGV6 (4) TRGV7 (4) TRGV8 (2) TRGV9 (3) TRGV10 (1) TRGV11 (1)	TRGJ1 (1) TRGJ2 (1) TRGJ3 (1)	TRGC	one locus Chrom. 2	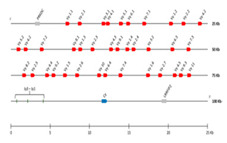 *Gallus gallus*	Liu et al., [69];
	Duck	TRGV1 (5) TRGV2 (2) TRGV3 (3) TRGV4 (1) TRGV5 (1) TRGV6 (1)	TRGJ1 (1) TRGJ2 (1) TRGJ3 (1) TRGJ4 (1) TRGJ5 (1)	TRGC	one locus no assigned	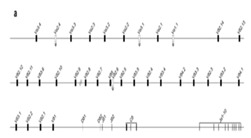 *Anas platyrynchos*	Yang et al., [70];
**FISHES**	Shark	TRGV1 (1) TRGV2 (1) TRGV3 (1) TRGV4 (1) TRGV5 (1)	TRGJ1 (1) TRGJ2 (1) TRGJ3 (1)	TRGC	one locus no assigned	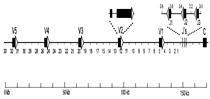 *Carcharhinus plumbeus*	Chen et al., [71];
	Atlantic salmon	TRGV1 (4) TRGV2 (3)	TRGJ1 (1) TRGJ2 (1) TRGJ3 (1) TRGJ4 (1) TRGJ5 (1)	TRGC1 (1) TRGC2 (1) TRGC3 (1) TRGC4 (1) TRGC5 (1)	two loci no assigned	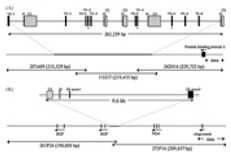 *Salmo salar*	Yazawa et al., [73];
**REPTILES**	Alligator	TRGV1 (1) TRGV2 (1) TRGV3 (1) TRGV4 (1) TRGV5 (6) TRGV6 (3) TRGV7 (1) TRGV8 (2) TRGV9 (1) TRGV10 (1)	TRGJ1 (1) TRGJ2 (1) TRGJ3 (1) TRGJ4 (1) TRGJ5 (1) TRGJ6 (1) TRGJ7 (1) TRGJ8 (1) TRGJ9 (1)	TRGC	one locus no assigned	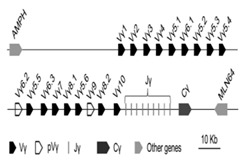 *Alligator sinensis*	Wang et al., [74];
**MARSUPIALS**	Opossum	TRGV1 (5) TRGV2 (1) TRGV3 (2) TRGV4 (1)	TRGJ1 (1) TRGJ2 (1) TRGJ3 (1) TRGJ4 (1) TRGJ5 (1) TRGJ6 (1) TRGJ7 (1)	TRGC	one locus Chrom. 6q	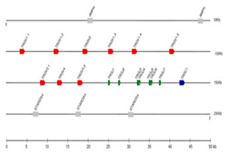 *Monodelphis domestica*	Parra et al., [75];

Legend: The numbers in brackets indicate the number of the genes.

In the Atlantic salmon (*Salmo salar*), two different TRG loci were identified [73]. Unlike the other outgroup species, the salmon TRG loci are organized in cassettes, each containing the basic V-J-C unit. The first locus spans 260 kb and contains five tandem-repeated cassettes, each of which consists of one to four *TRGV*, one *TRGJ*, and one *TRGC* genes. The only exception is the TRGJ3-C3, which is missing the 5′ end *V* genes. A total of 10 *TRGV* (7 functional genes belonging to two subgroups and 3 pseudogenes), 5 *TRGJ*, and 5 *TRGC* genes were found in Locus 1 (Table 1, Supplementary Figure S1D). Within Locus 2, a single V-J-C cassette was found, consisting of one gene for each type. Genomic comparison between the two TRG loci indicated that the duplication of these loci was not derived from whole-genome duplication in salmonids, but rather that it might be the result of a separate partial duplication event [73]. 

The six *TRGC* genes are all potentially functional and present a different structure. The *TRGC2*, *TRGC3*, and *TRGC5* genes are organized into three exons (EX1, EX2, and EX3), while the *TRGC1* gene has four exons, including two EX2 (EX2A and EX2B). The *TRGC4* and *TRGC6* genes have only two exons (EX1 and EX3), and are missing Exon 2 (EX2), which codes for the connecting region. Expression study within Locus 1 has revealed that the V genes located within each cassette preferentially recombine with the J genes in their own cassette. However, several cDNA clones show unique rearrangements of V genes that are rearranged by skipping over cassettes [73]. This form of rearrangement may be a mechanism for generating more potential diversity for antigen recognition. 

The genomic organization of the TRG locus in *Alligator sinensis* was recently defined based on the analysis of BAC clones [74]. The TRG locus of *Alligator sinensis* spans 115 kb and consists of a J-C cluster (nine *TRGJ*–one *TRGC*) (Table 1, Supplementary Figure S1E) preceded by 18 *TRGV* genes belonging to 10 subgroups. Two of these 18 *TRGV* genes are pseudogenes, as they contain in-frame stop codons. The *TRGC* gene presents the three-exon basic structure. *Amphiphysin* (*AMPH*) is found at the 5′ end of the locus, and *Related to steroidogenic acute regulatory protein D3-N-terminal like* (*STARD3NL* or *MLN64* in Supplementary Figure S1E) with an inverted orientation is found at the 3′ end of the locus. 

The organization of the conventional TRG locus in the opossum *Monodelphis domestica* is highly conserved, with a similar complexity to that of eutherians (placental mammals) (Table 1, Supplementary Figure S1F). Only a single TRG locus has been identified, and this corresponds to the locus mapped previously to Chromosome 6q [76]. *AMPH* and the *STARD3NL* are found at the 5′ and 3′ end of the TRG locus, respectively. From the 5′-most *TRGV* gene to the 3′ untranslated region (UTR) of the single *TRGC* gene, the opossum TRG locus spans only approximately 90 kb. There are nine *TRGV* genes present in the opossum and these are divided into four subgroups [75]. All *TRGV* genes appear to be functional, and have been found to be expressed in the thymus. In the J-C-cluster (seven *TRGJ*–one *TRGC*), the opossum *TRGC* is encoded by three exons. Exon 1, which encodes the C-γ domain, contains a single N-glycosylation site at position IMGT N101 [60]. An unusual characteristic described in marsupials is the absence of the second cysteine (2nd-CYS), C104, required for the formation of the intrachain disulfide bond in Exon 2 [60]. All non-eutherian mammals (monotremes and marsupials) lack 2nd-CYS C104, a loss apparently due to independent mutations in marsupials and monotremes [77].

## 5. The TRG Locus in Cetartiodactyla and Carnivora: The “Gene Cassette” Model

### 5.1. Genomic Organization of Ovine, Bovine, Camel, and Dolphin TRG Loci 

**Figure 2:**
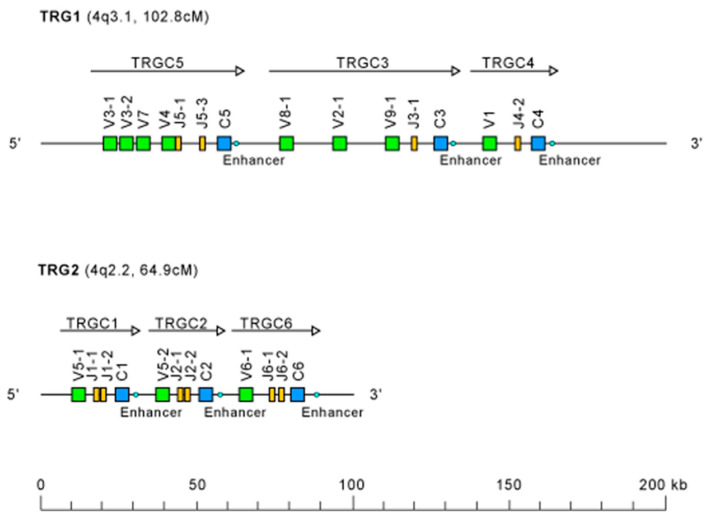
Sheep (*Ovis aries*) TRG locus representation, reproduced with permission from IMGT^®^ (http://www.imgt.org). The *TRG* genes are organized in two loci on Chromosome 4. Colors are according to IMGT color menu for genes (https://www.imgt.org/IMGTScientificChart/RepresentationRules/colormenu.php#LOCUS). The boxes representing the genes are not to scale. Exons are not shown. Arrows indicate the TRGC cassettes and the transcriptional orientation of their genes.

In this review of the evolution of the TRG locus in mammals, the unexpected finding described by Massari et al. [78] is certainly relevant. In the paper, the authors reported the cytological mapping of two sheep phage λ genomic clones containing two distinct TRGC sequences. FISH experiments on sheep metaphases highlighted the presence of two TRG paralogous loci separated by at least five chromosomal bands on Chromosome 4 (Figure 2). 

One locus, named TRG1, is located on 4q3.1 within a region of homology with the human Chromosome 7p14, where the TRG locus maps [79]. The other locus, TRG2, mapping on 4q15–22, is not included in the region of synteny with respect to humans, thus appearing to be peculiar to sheep. This finding represented the first case in mammals in which a TR locus was not found in a single chromosomal region. In humans, only one case of extensive interchromosomal duplication has been reported. It regards the TRB locus, where several *TRBV* genes moved from the main TRB locus on Chromosome 7q34 to Chromosome 9p21, causing the emergence of an orphan locus [80,81]. Conversely, expression assays [82,83,84] have shown that both sheep TRG loci are functional.

The subsequent availability of BAC clones containing TRG1 and TRG2 locus sequences [85] made it possible to extend FISH experiments to goat, cattle, and river buffalo. These studies located TRG2 loci on the homologous chromosome band of cattle and goat 4q22 and river buffalo 8q17 [86], showing that the presence of the paralogous TRG2 locus is typical of Bovidae.

The genomic structure of both TRG loci in sheep (Figure 2) highlights the peculiarity of the organization of these loci, consisting in a set of six (three for each locus) closely related “cassettes”, each containing the basic structure V–J–J–C unit arranged in the same transcriptional orientation [84]. All the J-J-C regions are delimited at their 5’ end by promoters for germline transcription containing STAT motifs, which control the local recombinational accessibility [87], and at their 3′ end by enhancer-like elements, which govern the general recombinational accessibility [88,89]. Preliminary expression studies have shown that each *TRGV* gene preferentially rearranges with the *TRGJ* genes of its cassette and, after transcription, the V-J region is spliced to the relevant *C* in mature transcripts [82,83]. The isolation of five TRG1 and two TRG2 BAC clones, their subcloning in plasmid vectors, and the sequencing of their inserts allowed the authors to obtain contiguous genomic sequences spanning 158.8 kb for TRG1 and 95.0 kb for TRG2 [89]. In Table 2 and Figure 2, the overall organization of the ovine TRG1 and TRG2 loci is shown. The entire TRG1 locus encompasses three cassettes, TRGC5, TRGC3, and TRGC4, named according to the constant genes [82,83] and following the recommendations of the IMGT Nomenclature Committee. Similarly, the entire TRG2 locus, consisting of TRGC1, TRGC2, and TRGC6 cassettes, is schematically represented. Comparative genomics and evolutionary analyses of the sheep TRG sequences support the idea that the TRGC5 cassette resembles the ancestral one, which underwent the first duplicative event that led to the birth of the two TRG loci [89]. Overall, six *TRGV* genes are located in the TRGC5 cassette and three in the TRGC3, while all the other cassettes consists of a single gene. The sheep *TRGV* genes belong to eleven subgroups, defined by a percentage of identity less than 75% for the V-region at the nucleotide level [44]. All sheep V subgroups consist of only one member gene, except the TRGV3 and the TRGV5 subgroups which have two genes each. Only two *TRGV* genes and three *TRGJ* genes are pseudogenes. 

With regard to the *TRGC* genes, all sheep genes are functional and, studied in detail, show a great structural diversity [90]. Exon 1 (EX1) is similar in all the *TRGC* genes and encodes the C-γ domain, which contains the two conserved 1st-CYS 23 and 2nd-CYS 104 required for the intra-chain disulfide and N-glycosylation sites N-X-S/T at positions IMGT N13, N83 (*TRGC4*), and N84.1 (*TRGC5* and *TRGC6*), according to the IMGT unique numbering for C-DOMAIN [60]. The connecting region (CO), which contains a single conserved cysteine that forms the interchain disulfide bond with the TRDC chain, differs in size between the sheep *TRGC* genes. The size differences result from a different number of exons encoding the CO: one exon (EX2A) for the *TRGC1* and *TRGC5* genes, two exons (EX2A and EX2C) for the *TRGC3* gene, and three exons (EX2A, EX2B, and EX2C) for the *TRGC2*, *TRGC4*, and *TRGC6* genes. Furthermore, the EX2A exon of all *TRGC* genes (excluding the *TRGC5* gene) contains a TTE(K)P(S)P motif in single copy or, for *TRGC2*, in triplicate. Finally, all *TRGC* genes have an almost identical Exon 3 (EX3) encoding the last part of the connecting region, the transmembrane region, and the cytoplasmic region (https://www.imgt.org/IMGTrepertoire/Proteins/protein/sheep/TRG/TRGC/Sh_TRGCallgenes.html).

The gene organization of the bovine TRG loci has been also determined [91,92]. Similarly to the ovine organization, the structure of the two bovine TRG loci consists of corresponding tandem-repeated V-J-J-C cassettes at each locus (Table 2 and Table 3, Supplementary Figure S2A).

To facilitate comparative studies between ruminants, and on the basis of their high sequence identity [89], gene names identical to those of sheep were assigned to the bovine TRGC cassettes by the IMGT Nomenclature Committee [93]. However, differences in the genomic organization with respect to the sheep loci can be observed, as summarized in Table 3. The bovine TRG1 locus is 178 kb long and includes an extra cassette (TRGC7) which, however, appears not to be functional because the *TRGC7* gene is a pseudogene and the *TRGJ* genes are absent [92]. Moreover, the incomplete nature of the bovine TRGC5 cassette (Supplementary Figure S2A) may justify the absence of the *TRGV11* gene and the presence of two more *TRGJ5* genes with respect to sheep (Table 3), while gene duplications involving the bovine *TRGV8* and *TRGV9* gene subgroups have occurred (Table 2, Supplementary Figure S2A and Table S2B). Conversely, the bovine TRG2 locus spans 103 kb and it appears to be more similar to the corresponding ovine locus except for the duplication of the *TRGV6* gene. The bovine *TRGC* genes present a structural diversity similar to that of the sheep genes (Table 3). In particular, the *TRGC5* has the classic three exons (EX1, EX2A, and EX3); TRGC3 and the *TRGC7* pseudogene have four exons (EX1, EX2A, EX2C, and EX3); all the others have five exons (EX1, EX2A, EX2B, EX2C, and EX3). A TT(A)EPP motif has been found in *TRGC4, TRGC2*, and *TRGC1* in single, duplicate, or quadruplicate form, respectively (https://www.imgt.org/IMGTrepertoire/Proteins/protein/Btaurus/Bt_TRCallgenes.html). In this species, expression analyses have also shown that V-J rearrangements among genes are favored within the same TRG cassette [91,92,94,95]. 

Comparative genomics in ruminants (*Ovis aries* and *Bos taurus*) highlights the fact that requirements related to immunoprotective functions, including the first defensive barrier in the epithelia of the digestive tract, are likely to have induced a sort of functional genome variability within TRG loci. As a consequence, the large number of *TRGV* genes, due to the reiterated duplications of TRG gene cassettes within the locus, increase the number of rearrangement events, which in turn produced transcripts with highly diversified variable domains [89].

Only recently, the identification of the 5′ and 3′ boundary genes (defined as IMGT 5′ and 3′ bornes), *AMPH* and *STARD3NL*, allowed the complete TRG locus in all its parts to be established in dromedary species [96]. The dromedary TRG locus is single. Starting from the first *TRGV11* gene at its 5′ end, and ending with *TRGC2* gene at its 3′ end, it spans about 105 kb and, similarly to each of the two TRG loci in sheep, it is organized into three V-J-J-C cassettes [97]. The distance between the *STARD3NL* gene and the last exon of the *TRGC2* gene is 8725 bp and in this portion no genes are present. The cassettes are classified as TRGC1, TRGC2, and TRGC5 [96] (Table 2, Supplementary Figure S2B). 

The ancestral nature of the TRGC5 cassette is highlighted by the high structural correspondence and the close phylogenetic relationship with the sheep cassette genes, with the exception of the presence of only one *TRGV3* gene and the functionality of the *TRGV11* and *TRGV10* genes in the dromedary cassette. As in sheep and bovine genomes, the *TRGC5* gene consists of three exons (EX1, EX2, and EX3). There are three *TRGJ* genes in the dromedary, as in Bovidae. Finally, the extensive collinearity shared between the entire dromedary TRGC5 cassette sequence and the corresponding cassette of the ovine TRG1 locus further confirmed the “ancient cassette” characteristic of the latter [96]. 

Only one *TRGV* gene is present in the other dromedary TRGC cassettes, whereas, the *TRGC1* and *TRGC2* genes, encoded by five exons, consist of three EX2, as observed in the sheep *TRGC2*, *TRGC4*, and *TRGC6* genes [97].

The total number of dromedary *TRGV* germline genes is certainly lower compared to that of sheep and cattle, and the TRG chain diversity due to the potential gene rearrangements is therefore more limited. However, cDNA sequencing clearly revealed that besides the combinatorial diversity and the introduction of N region diversity typical of all known *IG* and *TR* genes, a further mechanism enhances the TRG diversity in *Camelus dromedarius*. In line with previous reports [97], more recent studies [35,98] have provided direct evidence that somatic hypermutation (SHM) heavily contributes to the expansion of the γδ TR repertoire even in the absence of functional reiterated genome duplications. The frequency of mutations observed in the V-γ domain was comparable with that found in targeted genes in AID-induced T lymphomas [99], rearranged shark TRGV [71], and dromedary TRDV regions [100]. Previously, somatic hypermutation had been completely demonstrated only for *IG* genes in the B cells of higher vertebrates in order to produce antibodies with higher affinity. In contrast, in the dromedary as well as in sharks, the purpose of the somatic mutations in V-γ domains is to generate a more diverse repertoire of receptors. 

Finally, the dolphin TRG locus is the smallest and simplest of all mammalian loci studied to date [38]. It spans only 48 kb and its genes are arranged in a pattern comprising two *TRGV* belonging to two distinct subgroups, three *TRGJ* genes and a single *TRGC* gene (Table 2, Supplementary Figure S2C). A closer inspection of the shared dolphin and sheep genes revealed that the dolphin *TRGC* gene possesses a single small Exon 2 (EX2) similar to the sheep TRGC5 EX2. The dot-plot matrix of dolphin TRG and sheep TRG1 loci genomic comparison displays a remarkable consistency of the identity diagonals from the sheep *TRGV11-1* gene to the *TRGC5* gene, with a remarkable compactness of the three *J* genes [38]. The overall organization of the dolphin TRG locus is reminiscent of the typical single-cassette structure of artiodactyls [89], with a small number of genes. However, an evolutionary correlation can be also found between the two dolphin *TRGV* genes and the human *TRGV9* and *TRGV11* genes, as well as between the dolphin J-J-J-C region and the human J-J-J-C1 region [38]. Dolphin TRG-chain expression analysis from the blood and skin of unrelated subjects demonstrated that the two *TRGV* and three *TRGJ* genes were used in every possible combination, although a bias towards some transcripts (TRGV1-TRGJ2 and TRGV2-TRGJ3) was noted. Furthermore, about half of the transcripts using *TRGV2* were unproductive due to the presence of stop codons in CDR3. The percentage values of the productive/unproductive rearrangements were similar for both cDNA and genomic clones, and in the same and different individuals, in contrast to what is usually observed (the percentage of unproductive rearrangements being lower in cDNA, due to nonsense-mediated decay of RNA). The authors argued that the occurrence of clonotypes shared by different individuals living both in marine and in artificial marine “habitats”, and previously described as “convergent recombination” [101], could be in fact strictly related to the biased V-J rearrangement events.

A comparable preferential usage of the *TRGJP* (localized between *TRGJP1* and *TRGJ1*) and the predominant expression of TRGV9-JP-C1 chain paired with a TRDV2-D-J-C in humans may be related to promoter characteristics [102]; thus, the same observation in both dolphins and humans supports an accurate determination of the *TRGJ* gene usage. As a consequence, the high frequency of TRGV1-J2/TRDV1-D1-J4 productive rearrangements in dolphins may represent a situation of oligoclonality comparable to that found in human TRGV9-JP/TRDV2-D-J T cells [103]. 

### 5.2. Genomic Organization of Canine and Feline TRG Loci 

Table 2 and Supplementary Figure S2D show the overall organization of the dog TRG locus, which encompasses eight cassettes, named according to the constant genes. All TRG cassettes lie in the same transcriptional orientation, are closely spaced, and contain the basic recombinational unit V-J-J-C, except for the last cassette, J-J-C, which lacks the *V* gene and occupies the 3′ end of the locus. The limit dividing the eight cassettes from each other is approximately given by a space ranging from 10 to 18 kb (from the last exon of each *C* gene to the L-PART1 of the *V* gene of the downstream cassette), except for one of about 35 kb between Cassettes 6 and 7. The *AMPH* and *STARD3NL* genes flank, respectively, the 5′ and 3′ ends of the TRG locus which spans 460 kb. There are 16 *TRGV* genes assigned to seven subgroups. Four subgroups (TRGV2, TRGV3, TRGV5, and TRGV7) are multimembers with three or four genes; the other subgroups have only one gene member. The germline configuration and the exon–intron organization of the eight *TRGC* genes (*TRGC1*–*TRGC8*) has been well analyzed [104]. Six of them (*TRGC2* to *TRGC5*, T*RGC7*, and *TRGC8*) are functional, whereas *TRGC1* is an open reading frame (ORF) and *TRGC6* is a pseudogene. The first exon (EX1) encodes the C-γ domain, which comprises 110 amino acids or is slightly shorter (105 aa for *TRGC1*, 109 aa for *TRGC2* and *TRGC4*). The first part of the connecting region is encoded by one or two exons (EX2A and/or EX2B), a situation reminiscent of the artiodactyl *TRGC* genes. Thus, the canine *TRGC2*, *TRGC3*, and *TRGC4* genes have both EX2A and EX2B exons, whereas the *TRGC1*, *TRGC6*, *TRGC7*, and *TRGC8* genes have only a single EX2B exon, and *TRGC5* has a single EX2A exon. The remaining part of the connecting region (CO), the transmembrane region, and the cytoplasmic region are encoded by EX3 (https://www.imgt.org/IMGTrepertoire/Proteins/protein/dog/TRG/TRGC/Cf_TRGCallgenes.html). The reiterated cassette duplication in the canine TRG locus resulted in a total of 40 genes, with 21 of them functional and 19 pseudogenes or ORFs. On the other hand, the low ratio of functional genes to the total number of canine *TRG* genes (23/40), suggests that there is no correlation between the extensive duplications of the cassettes and a need for new functional genes. In contrast to the bovine and ovine TRG loci, the extensive duplication of the TRG cassettes does not seem to match a real need of the adaptive immune response.

The *Felis catus* TRG locus spans approximately 260 kb in the pericentromeric region of Chromosome A2. The 5′ IMGT borne is *AMPH* and the 3′ IMGT borne is *STARD3NL* (Table 2, Supplementary Figure S2E). The TRG locus contains 30 genes. There are 12 *TRGV* genes (six functional and six pseudogenes) assigned to six subgroups, 12 *TRGJ* genes (four functional, two ORFs, and six pseudogenes), and 6 *TRGC* genes (four functional and two pseudogenes) arranged into five complete and one incomplete V-J-(J)-C cassettes [27]. The feline *TRGV* genes belong to six subgroups, two of which have four members (TRGV2 with four functional genes, TRGV5 with one functional and three pseudogenes) and the four others with a single member each; *TRGV7* is the only functional one, *TRGV6* is a pseudogene owing to two stop codons in the V region, and *TRGVA* and *TRGVB* are degenerate pseudogenes. The canine TRGV1 and TRGV3 subgroup orthologs are absent in the cat genome [104]. The 12 feline *TRGJ* genes were designated based on the cassette they belong to. There are four functional *TRGJ* genes, two ORFs, and six pseudogenes. Each TRGC region is encoded by three exons (EX1, EX2A or EX2B, and EX3) and all are functional except for *TRGC5* and *TRGC6* due to frameshifts in EX1 and EX3, respectively [27]. 

The feline TRG locus most closely resembles that of the dog, which has 8 V-J-(J)-C cassettes [http://www.imgt.org/ IMGTrepertoire/LocusGenes]. The fact that Cassettes 4 and 5 are in an inverted orientation in the cat, despite a high homology to dog *V* genes, suggests that the inversion likely occurred after speciation. 

Expression data have shown that the genomic cassette organization of the cat *TRG* genes may favor physical *V* and *J* proximity in the rearrangement, and that the greater effectiveness of this physical proximity in pursuing a strong gene expression depends on the functionality of the constant gene with which it is associated [27].

**Table 2:** Genomic organization and gene content of the TRG locus in Cetartiodactyla and Carnivora.

		TRGV Genes	TRGJ Genes	TRGC Genes	Chromosomal Localization	Miniature Locus	References
**CETARTIODACTYLA**	Ovine	TRGV1 (1) TRGV2 (1) TRGV3 (2) TRGV4 (1) TRGV5 (2) TRGV6 (1) TRGV7 (1) TRGV8 (1) TRGV9 (1) TRGV10 (1P) TRGV11 (1P)	TRGJ1 (2) TRGJ2 (2) TRGJ3 (2) * TRGJ4 (2) * TRGJ5 (3) ° TRGJ6 (2)	TRGC1 (1) TRGC2 (1) TRGC3 (1) TRGC4 (1) TRGC5 (1) TRGC6 (1)	Two loci Chrom. 4q31 Chrom. 4q22	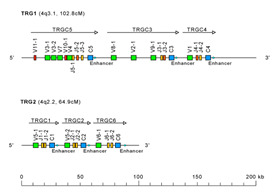 *Ovis aries*	Massari et al., [78]; Miccoli et al., [85]; Vaccarelli et al., [84,89];
	Bovine	TRGV1 (1) TRGV2 (1) TRGV3 (2) TRGV4 (1) TRGV5(1+1O) TRGV6 (2) TRGV7 (1) TRGV8 (4) TRGV9 (2) TRGV10 (1)	TRGJ1 (2) TRGJ2 (2) TRGJ3 (1) TRGJ4 (2) TRGJ5 (1) TRGJ6 (1)	TRGC1 (1) TRGC2 (1) TRGC3 (1) TRGC4 (1) TRGC5 (1) TRGC6 (1) TRGC7 (1P)	Two loci Chrom. 4q31 Chrom. 4q1.5-2.2	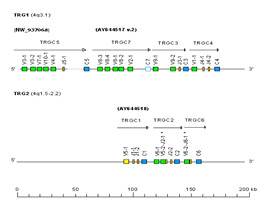 *Bos taurus*	Antonacci et al., [86]; Conrad et al., [92];
	Camel	TRGV1 (1) TRGV2 (1) TRGV3 (1) TRGV4 (1O) TRGV7 (1) TRGV10 (1) TRGV11 (1)	TRGJ1 (2) * TRGJ2 (2) * TRGJ5 (3) °	TRGC1 (1) TRGC2 (1) TRGC5 (1)	One locus Chrom. 7 q11.12	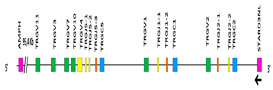 *Camelus dromedarius*	Vaccarelli et al., [97]; Antonacci et al., [96];
	Dolphin	TRGV1 (1) TRGV2 (1)	TRGJ1 (1) TRGJ2 (1) TRGJ3 (1)	TRGC	One locus no assigned	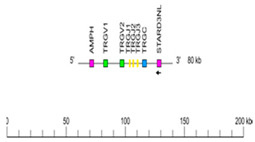 *Tursiops truncatus*	Linguiti et al., [38];
**CARNIVORA**	Canine	TRGV1(1P) TRGV2(4) TRGV3 (3P) TRGV4 (1O) TRGV5(3) § TRGV6(1P) TRGV7(3) #	TRGJ1 (2) * TRGJ2 (2) * TRGJ3 (2) * TRGJ4 (2) * TRGJ5 (2) TRGJ6 (2) TRGJ7 (2) * TRGJ8 (2) *	TRGC1 (1O) TRGC2 (1) TRGC3 (1) TRGC4 (1) TRGC5 (1) TRGC6 (1P) TRGC7 (1) TRGC8 (1)	One locus Chrom. 18	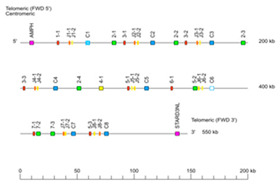 *Canis lupus familiaris*	Massari et al., [104]; Martin et al., [39];
	Feline	TRGV2(4) TRGV5(4) $ TRGVA(1P) TRGVB(1P) TRGV6(1P) TRGV7(1)	TRGJ1 (2) * TRGJ2 (3) ^ TRGJ3 (2) * TRGJ4 (1P) TRGJ5 (2) * TRGJ6 (2)	TRGC1 (1) TRGC2 (1) TRGC3 (1) TRGC4 (1) TRGC5 (1P) TRGC6 (1P)	One locus Chrom. A2	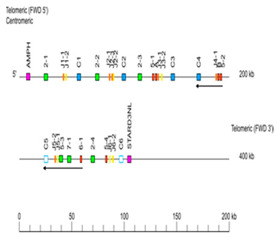 *Felis catus*	Radtanakatikanon et al., [27];

Legend: TRGV: § (1F+2P); # (2F+1P); $ (1F+3P); TRGJ: * (1F+1P); ° (2F+1P); ^ (1F+2P); P = pseudogene, O = ORF. The numbers in brackets indicate the number of the genes.

**Table 3:** Cassette comparison between *Ovis aries* and *Bos taurus*.

**TRG1 locus**	***Ovis aries* 4q3.1**			***Bos taurus* 4q3.1**		
Cassettes names	IMGT gene names	Nb of alleles	CDR-IMGT lengths	IMGT gene names	Nb of alleles	CDR-IMGT lengths
**TRGC5**	*TRGV11-1*	1 P	[8.6.4]			
	*TRGV3-1*	2 F	[8.7.4]	*TRGV3-1*	1 F, 1 (F)	[8.7.4]
	*TRGV3-2*	1 F	[8.7.5]	*TRGV3-2*	1 F, 1 (F)	[8.7.4]
	*TRGV7*	1 F	[8.6.6]	*TRGV7-1*	1 F, 1 (F)	[8.6.6]
	*TRGV10-1*	1P	[9.7.4]	*TRGV10-1*	1 F	[9.7.5]
	*TRGV4*	2 F	[8.4.5]	*TRGV4-1*	1 (F)	[8.4.x]
	*TRGJ5-1*	1 F	-	*TRGJ5-1*	1 F	-
	*TRGJ5-2*	1 P	-			
	*TRGJ5-3*	1 F	-			
	*TRGC5* (EX2A)	1 (F)	-	*TRGC5* (EX2A)	1 F, 2 (F)	-
(TRGC7)	*TRGV8-1*	1 F, 1 F	[3.8.4]	*TRGV8-3*	1 F, (1 F)	[3.8.5]
				*TRGV8-4*	1 F	[3.8.5]
				*TGRV8-1*	1 F	[3.8.5]
				*TRGV8-2*	1 F, (1 F)	[3.8.5]
	*TRGV2-1*	2 F, 1 (F)	[6.8.5] del81,82,83	*TRGV2-1*	1 F	[6.8.5] del81,82,83
				*TRGC7* (EX2A,2C)	1 P	-
**TRGC3**	*TRGV9-1*	1 F, 1 (F)	[6.8.4]	*TRGV9-1*	1 F	[6.8.5]
				*TRGV9-2*	1 F	[6.8.5]
	*TRGJ3-1*	1 F	-	*TRGJ3-1*	1 F, 1 (F)	-
	*TRGJ3-2*	1 P	-			
	*TRGC3* (EX2A,2C)	1 F, 1 (F)	-	*TRGC3* (EX2A,2C)	1 F, 1 (F)	-
**TRGC4**	*TRGV1* (L41)	2 F	[5.8.5]	*TRGV1-1* (L41)	1 F, 2 (F)	[5.8.5]
	*TRGJ4-1*	1 P	-	*TRGJ4-1*	1 F	-
	*TRGJ4-2*	1 F	-	*TRGJ4-2*	1 F	-
	*TRGC4* (EX2A,2B,2C)	1 (F)	-	*TRGC4* (EX2A)	1 F, 1 (F)	-
**TRG2 locus**	***Ovis aries* 4q2.1**			***Bos taurus* 4q1.5-2.2**		
Cassettes	IMGT gene names	Nb of alleles	CDR-IMGT	*Bos taurus*	Nb of alleles	CDR-IMGT
**TRGC1**	*TRGV5-1* (L41)	2 F	[5.8.5] del5-8	*TRGV5-1* (L41)	1 F, 4 (F)	[5.8.5] del5-8
	*TRGJ1-1*	1 F	-	*TRGJ1-1*	1 F	-
	*TRGJ1-2*	1 F	-	*TRGJ1-2*	1 F, 2 (F)	-
	*TRGC1* (EX2A)	1 F	-	*TRGC1* (EX2A,2B,2C)	1 F, 3 (F)	-
**TRGC2**				*TRGV6-1*	1 F, 1 (F)	[5.8.5] del16
	*TRGV5-2*	2 F	[5.8.5] del5-8	TRGV5-2*	10 (F)	[5.8.5] del5-8
	*TRGJ2-1*	1 F	-	TRGJ2-1*	1 F, 2 (F)	-
	*TRGJ2-2*	1 F	-	*TRGJ2-2*	1 F, 2 (F)	-
	*TRGC2* (EX2A,2B,2C)	1 F, 1 (F)	-	*TRGC2* (EX2A,2B,2C)	1 F, 4 (F)	-
**TRGC6**	*TRGV6-1*	1 F, 1 (F)	[5.8.5] del16	TRGV6-2*	1 F	[5.8.5] del16
	*TRGJ6-1*	1 F	-	TRGJ6-1*	1 F	-
	*TRGJ6-2*	1 F	-			-
	*TRGC6* (EX2A,2B,2C)		-	*TRGC6* (EX2A,2B,2C)	1 F, 1 (F)	-

Highlighted (in yellow) are sequence features found in both *Ovis aries* and *Bos taurus* TRG sequences (‘del’ followed by numbers indicate gap (unoccupied) positions according to the IMGT unique numbering for V-DOMAIN [58], ‘L41’ indicates that the CONSERVED-TRP 41 is replaced by a leucine (L) in the V-REGION. ‘EX2A’, ‘EX2A,2C’ and ‘EX2A,2B,2C’ indicate exon designation for *TRGC* gene with 1, 2 or 3 Exons 2, respectively.

## 6. The TRG Locus in Rodentia, Lagomorpha, and Primata

The *Mus musculus* TRG is located in a single position on Chromosome 13A2 and it occupies a region of about 200 kb, typically delimited by the *AMPH* and *STARD3NL* genes at the 5′ and 3′ ends, respectively (Table 4, Supplementary Figure S3A) [66,68,105,106,107]. The arrangement of the *TRG* genes resembles the Artiodactyla and Carnivora organization into cassettes, even if the number both of cassettes and of total genes are lower. Overall, the mouse locus comprises seven *TRGV* genes belonging to five subgroups, four *TRGJ* and four *TRGC* functional genes organized into four classical V-J-C cassettes. The first cassette, TRGC1, is the most extensive at 41 kb where, proceeding from 5′ to 3′, the TRGV7, TRGV4, TRGV6, and TRGV5 subgroup genes (one gene for each subgroup) are located. The TRGC3, TRGC2, and TRGC4 cassettes follow, all consisting of one *TRGV* and each belonging to a diverse subgroup, followed by one *TRGJ* and one *TRGC* gene. However, the TRGC3 cassette is not functional because of the *TRGC3*, while the entire TRGC2 cassette is inverted in the locus with respect to the other three cassettes. Enhancer elements that control the general accessibility of the region have been identified at the 3′ end of all V–J–C cassettes [108]. 

The mouse TRGC1, TRGC2, and TRGC3 chains have a short hinge domain while the TRGC4 chain is polymorphic with a large hinge region [67]. Analysis of the genomic organization of the mouse *TRGC* genes revealed that they are characterized by three exons (EX1, EX2, and EX3). However, the *TRGC4* gene can be constituted by one exon (EX2A) or, as in dog or artiodactyl *TRGC* genes [90,104], by two small exons (EX2A + EX2B), respectively 18 and 15 amino acids long, encoding the first part of the polymorphic hinge region of the TRGC4 chain (https://www.imgt.org/IMGTrepertoire/Proteins/protein/mouse/Mu_TRallgenes.html). 

If the dolphin TRG represents the smallest and simplest locus within the mammalian superorder Laurasiatheria (Cetartiodactyla/Perissodactyla/Carnivora), the rabbit (*Oryctolagus cuniculusis*) TRG can be considered the smallest and simplest locus identified to date within the Eurarchontoglires (Primate/Lagomorpha/Rodentia). In fact, the rabbit TRG locus spans about 70 kb and contains 11 *TRGV* genes upstream of 2 *TRGJ* genes and 1 *TRGC* gene (Table 4, Supplementary Figure S3B) [109]. Hence, the *TRG* gene arrangement in the rabbit is comparable to the cluster organization described earlier in most of the outgroups. A possible enhancer element has been identified about 8 kb downstream of the last exon of the *TRGC* gene. The *AMPH* gene is 13 kb upstream of the first *TRGV* gene, while *STARD3NL* is 1 kb downstream of the enhancer-like region, in an inverted transcriptional orientation. The *TRGV* genes (eight functional and three ORFs) are classified in four subgroups: the TRGV1 subgroup comprises eight members, and the TRGV2, TRGV3, and TRGV4 subgroups only have one member each. A high level of nucleotide identity between rabbit and human TRGV1 subgroup genes, both located in the 5′ part of the respective locus, has been found. The only *TRGC* gene consists of four exons, with two exons (EX2A and EX2B) that encode for the first part of the connecting region.

In contrast to the evolutionary conservation of the TRB and TRA/TRD loci, dynamic evolution of the TRG locus is demonstrated within the primate lineage. More recently, the TRG locus has been determined in four nonhuman primate species: in great apes, *Pan troglodytes* and *Pongo pygmaeus*; in *Macaca mulatta* (an Old World monkey) (Table 4, Supplementary Figure S3C); and in *Callithrix jacchus* (a New World monkey) [110]. 

Although the structure of the locus is still maintained with respect to the human locus, substantial diversification can be observed. In particular, the TRGV1 subgroup region is highly dynamic; it maintains a flanking position in all species, but the genes have undergone substantial duplications/deletions in which the orthology between genes has become less clear. For example, in the *M. mulatta* locus (Table 4, Supplementary Figure S3C), the number of *TRGV1* genes is more contained compared to human, with five genes of which only three are functional. While the first three genes show an evident orthology with respect to the first three human genes, the last ones seem to have been generated by a recent species-specific duplication [110]. 

In contrast, the TRGV9-TRGV10-TRGV11 region, which retains a central position in the locus, appears to be conserved with a high level of homology between the primate species, although *TRGV11* was not found in *C. jacchus*, and *TRGV10* is functional in *Pan troglodytes*. Two *TRGC* genes are present in all species, while the number and distribution of *TRGJ* varies, as in the case of *M. mulatta* where, unlike the human locus, two *TRGJ* genes are before the *TRGC1* gene and three *TRGJ* (one is not functional), are upstream of the *TRGC2* gene. In *M. mulatta* the *TRGC1* and *TRGC2* genes have three and five exons, respectively, and the presence of polymorphisms has not been determined.

## 7. Phylogenetic Analysis Highlights a Diverse Mode of Evolution of the V, J, and C Subfamily Genes

The rearranged *V*-*D*-*J* and *V*-*J* genes encode the V-β and V-α, or the V-δ and V-γ domains, respectively, which form the antigen-binding site of the αβ or γδ TR receptor, while the *C* genes encode the C-region which comprises the C-domain (C-β and C-α, or C-δ and C-γ), the connecting region, the transmembrane region which anchors each receptor chain in the membrane, and a very short (absent for the delta chain) cytoplasmic region [1]. CD3 proteins are associated to the αβ or γδ TR for the signal transduction. Thus, the TcR of the T cells, constituting the TR + CD3 coreceptors, is the equivalent of the BcR of the B cells, constituting the IG and CD79 coreceptors.

**Figure 3:**
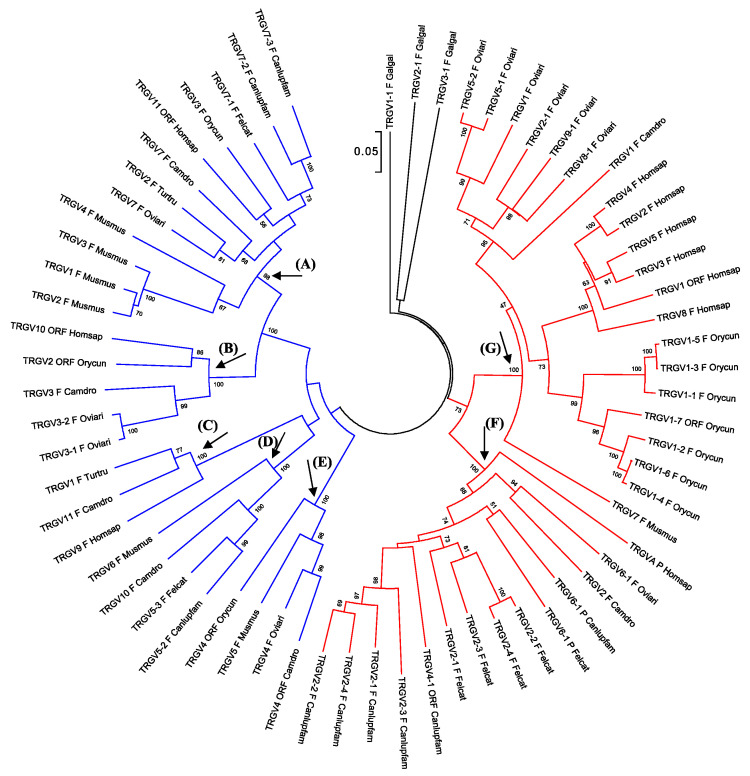
Evolutionary relationships of the eutherian mammalian *TRGV* genes. The TRGV as well as the TRGJ (Figure 4) and TRGC (Figure 5) gene sequences used for the phylogenetic analysis were retrieved from the IMGT^®^ database (IMGT Repertoire, http://www.imgt.org) if annotated, or from the GenBank database using the accession numbers reported in the reference articles listed in Table 2 and Table 4. Chicken (Galgal) *TRG* genes were used as the outgroup [66]. For simplicity, we included in the analysis the *TRGV* genes belonging to a single species for each mammalian suborder in which the genomic organization of the TRG locus has been inferred. One member gene for each of the chicken (Galgal) TRGV subgroups was used as an outgroup. Multiple alignments of the V-region nucleotide sequences of functional genes and in-frame pseudogenes were carried out with the MUSCLE program [111]. The evolutionary analyses were conducted in MEGA7 [112]. We used the neighbor-joining (NJ) method to reconstruct the phylogenetic tree [113]. The percentage of replicate trees in which the associated taxa clustered together in the bootstrap test (100 replicates) is shown next to the branches [114]. The trees are drawn to scale, with branch lengths in the same units as those of the evolutionary distances used to infer the phylogenetic trees. The evolutionary distances were computed using the p-distance method [115], and the units are the number of base differences per site. The analysis involved 66 nucleotide sequences. All positions containing gaps and missing data were eliminated. There were a total of 176 positions in the final dataset. Monophyletic groupings described in the text are indicated by capital letters. The blue and red branches of the tree highlight two major groupings of the mammalian genes. The IMGT six-letter standardized abbreviations for species (Homsap (human), Musmus (mouse), Felcat (cat), Oviari (sheep), Camdro (dromedary), Turtru (dolphin), Orycun (rabbit)) and nine-letter abbreviations for subspecies (Canlupfam, dog) taxa are used.

The different roles of the types of genes that make up the receptor seems to influence each one’s way of evolving. Generally, the genes encoding the variable domain seems to follow evolutionary dynamics, shared among species, that preserve the sequence of the orthologous genes; whereas, the evolution of the *C* genes is reflective of the phylogenetic history of each species. This observation (or consideration) comes from analysis of the TRB and TRA/TRD loci, which retain a conserved general gene arrangement and, although these regions appear to have been evolving dynamically in each eutherian mammalian species, a gross order of genes is still maintained. As a matter of fact, phylogenetic analysis conducted on the *TRBV* and *TRAV* genes showed that the *V* genes from different mammals intermingle rather than forming separate clades, proving that duplications of ancestral genes followed by diversification is the major mode of evolution [26,27,35,37,40]. This rule also fits also the *TRG* genes, despite the structure of the TRG locus differing considerably across species. Indeed, the first phylogenetic investigations involving mammalian *TRG* genes showed that the sheep *TRGV* genes also form groupings with human and bovine genes [85]. Subsequent studies led over time to the identification of the genomic organization of the TRG locus in many other mammals such as rabbits, dogs, camels, dolphins, and cats, and confirmed the first evolutionary indication, despite the discovery of a great diversity of the gene arrangement within the TRG region of the various species [27,38,96,109]. The tree shown in Figure 3 recapitulates the evolutionary relationship between the mammalian *TRGV* genes by combining pre-existing phylogenetic data. 

Despite the dynamic evolution of each TRG locus, the *TRGV* genes of the different species intermingle with each other to form monophyletic groups (A–G) rather than separate species-specific clades. Indeed, each branch groups corresponding genes (or gene subgroups/subfamilies) of all species with a clear orthology, irrespective of their genomic organization, indicating their occurrence from a common ancestor and a strong selective pressure to maintain their function. For instance, Branch A groups corresponding genes which have been preserved in all species of mammals as a result of a strong functional constraint. Other monophyletic clades (B–G) group corresponding genes present in some but not in all species in accordance with the birth-and-death model of multigene family evolution, which explains that some duplicated genes are retained in the genome for a long time, while others are deleted or become pseudogenes. This evolutionary model also explains the emergence of new genes that have undergone substantial diversification through species-specific duplication events, as indicated in the tree by species-specific clustering of the genes (especially in the G and F clades). 

Moreover, looking at the tree as a whole, two major groupings of the mammalian genes are clearly distinguishable (the blue and red branches of the tree in Figure 3). The blue branch groups genes exhibiting a conserved nature across species with a clear correspondence between genes, whereas the red part of the tree contains *TRGV* genes that have undergone substantial diversification through duplications within each species. For example, the blue branch includes all the artiodactyl *TRGV* genes belonging to the TRGC5 cassette, which has been shown to be the most evolutionarily ancient [89]. This cassette would have been duplicated to generate a second one from which the other artiodactyl TRGC cassette developed. The red part of the tree includes the *TRGV* genes of the second cassette and all its derivatives. As another example, in the blue part of the tree can be found the human *TRGV9*, *TRGV10*, and *TRGV11* genes, which have been proven to be conserved across the primate lineage and to occupy similar positions within the different TRG loci. Conversely, the human genes grouped in the red part of the tree have undergone a highly dynamic evolution, making their orthology with the nonhuman primate genes unclear (Figure 3). 

Overall, one can speculate that the *TRGV* genes present today in the different mammalian species derived from two different ancestors. The blue branch of the tree groups the genes derived from the oldest one, and they have mostly have maintained evolutionary stability, while, the red part consists of new repertoires of genes that evolved dynamically by duplication (and presumably deletion) even within closely related species.

The peculiar genomic organization of TRG loci in cassettes, clusters, or semi-clusters favors the physical proximity of *TRGV* and *TRGJ* genes and prompted investigation of the phylogenetic behavior of the *TRGJ* with respect to *TRGV* and *TRGC* genes.

**Figure 4:**
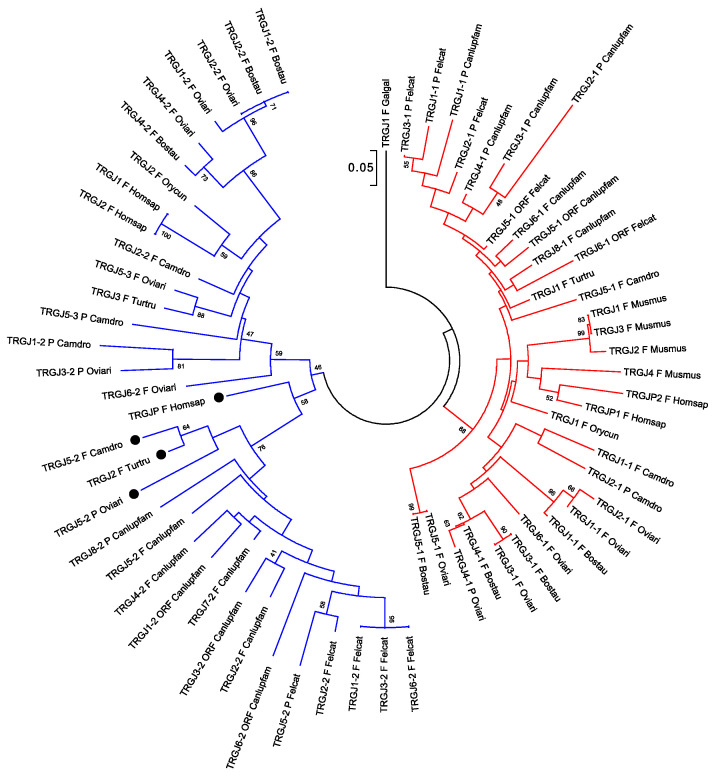
Evolutionary relationships of the eutherian mammalian *TRGJ* genes. The TRGJ coding sequences of all mammalian species were included in the tree. A chicken (Galgal) *TRGJ* gene was used as outgroup [66]. Multiple alignments of the gene sequences were carried out using the MUSCLE program [111]. The evolutionary analyses were conducted in MEGA7 [112]. We used the neighbor-joining (NJ) method to reconstruct the phylogenetic tree [113]. The percentage of replicate trees in which the associated taxa clustered together in the bootstrap test (100 replicates) is shown next to the branches [114]. The trees are drawn to scale, with branch lengths in the same units as those of the evolutionary distances used to infer the phylogenetic trees. The evolutionary distances were computed using the p-distance method [115] and the units are the number of base differences per site. The analysis involved 67 nucleotide sequences. All positions containing gaps and missing data were eliminated. There were a total of 39 positions in the final dataset. The C-proximal *TRGJ* genes are shown in red; the C-distal *TRGJ* genes are shown in blue. The *TRGJ* genes occupying the middle position of the J cluster formed by three genes within each own TRG locus are marked with a black circle. The IMGT six-letter standardized abbreviations for species (Homsap (human), Musmus (mouse), Felcat (cat), Bostau (bovine), Oviari (sheep), Camdro (dromedary), Turtru (dolphin), Orycun (rabbit)) and nine-letter standardized abbreviation for subspecies (Canlupfam, dog) taxa are used.

Evolutionary analyses conducted with the human, sheep, cattle, dolphin, and dromedary TRGJ sequences showed the clustering of the *TRGJ* genes into two main groups in relation to the physical position of each *J* gene within its own cluster with respect to the *TRGC* gene [38,88,96]. An updated version of the same evolutionary analyses performed with the addition of the TRGJ sequences of mammalian species representative of Carnivora, Rodentia, and Lagomorpha orders (Figure 4), confirmed the division of the *TRGJ* genes into two major monophyletic groupings. 

The red branch consists of all the *J* genes (paralogous genes within and between each species and orthologous genes between species) occupying the position closest to the constant gene (C-proximal) within the V-J-(J)-J-C clusters, as well as all the mouse *TRGJ* genes, which are single in each TRGC cassette (Figure 1A, Figure 2, and Supplementary Figures S2 and S3). In the blue branch, all the *J* (paralogous and orthologous) genes occupying the farthest position from the constant gene (C-distal) are present together with the *TRGJ* genes that are in the middle of the J cluster formed by three genes within the human, sheep, camel, and dolphin TRG loci (black circles in Figure 4). These genes form a paraphyletic group that differentiates the *TRGJ* genes of carnivores from those of other mammalian species. These data highlight an evident functional constraint of the *J* genes, which play a role in the recombination process and structurally contribute to the V domain of the receptor [2].

**Figure 5:**
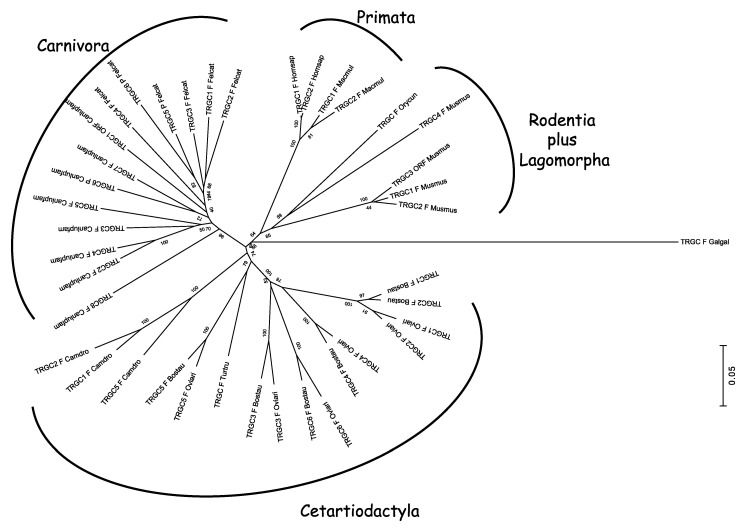
Phylogenetic relationships of the eutherian mammalian *TRGC* genes. The TRGC coding nucleotide sequences from the different eutherian mammals were combined in the same alignment. The chicken (Galgal) *TRGC* gene was used as outgroup [66]. Multiple alignments of the gene sequences were carried out using the MUSCLE program [111]. The evolutionary analyses were conducted in MEGA7 [112]. We used the neighbor-joining (NJ) method to reconstruct the phylogenetic tree [113]. The percentage of replicate trees in which the associated taxa clustered together in the bootstrap test (100 replicates) is shown next to the branches [114]. The trees are drawn to scale, with branch lengths in the same units as those of the evolutionary distances used to infer the phylogenetic trees. The evolutionary distances were computed using the p-distance method [115] and the units are the number of base differences per site. The analysis involved 38 nucleotide sequences. All positions containing gaps and missing data were eliminated. There were a total of 324 positions in the final dataset. The IMGT six-letter standardized abbreviations for species (Homsap (human), Musmus (mouse), Felcat (cat), Bostau (bovine), Oviari (sheep), Camdro (dromedary), Turtru (dolphin), Orycun (rabbit)) and nine-letter standardized abbreviations for subspecies (Canlupfam, dog) taxa are used.

In contrast to the intermingling of the *TRGV* and *TRGJ* genes from different species, the mammalian *TRGC* genes evolved in a species-specific manner, and the sequences form distinct clades consistent with the current phylogeny [88,90,96]. The tree shown in Figure 5 recapitulates the evolutionary relationships of the *TRGC* genes in the different eutherian mammalian species and highlights the species-specific grouping. 

Gene conversion seems to be the mechanism that has homogenized the TRGC sequences within each lineage (concerted evolution), maintaining a high level of similarity between the TRGC protein isotypes in each species. This conservation is constrained by structural and functional requirements. In fact, the constant portion of the TR does not bind antigen, while it must interact with monomorphic structures like the extracellular part of the TRD chain and the CD3 coreceptors; therefore, it plays an important role in signal transduction. 

However, orthology between multiple *TRGC* genes can be maintained in more closely related species, as in cattle and sheep. In contrast, dromedary *TRGC* genes group apart from the other Cetartiodactyla suborders, for which a common ancestor gene can be hypothesized.

## 8. Conclusions

The great plasticity of the adaptive T-cell receptor repertoire in vertebrates is mainly due to gene duplication and to somatic rearrangement during T-cell differentiation. Ohno postulated that gene duplication plays a major role in evolution in his book “Evolution by Gene Duplication” [116]. Here, we refer to duplication-driven evolution of *V*, *J*, and *C* genes of the TRG locus in mammals and discuss the correlation between its genomic organization and the possible modalities of duplication in organisms belonging to the superorders of Cetartiodactyla (Ruminantia/Tylopoda/ Cetacea) and Carnivora in comparison with Rodentia, Lagomorpha, and Primata and with outgroups.

In this regard, a careful look at the genomic organization of TRG inside the outgroup (birds, fishes, reptiles, and marsupials) locus reveals that the duplications have affected (involved) the *V* and *J* genes individually by creating clusters. Hence, duplications have occurred in the region of the *TRGJ* genes with the main objective of obtaining a high number (seven in the marsupials and nine in the alligator) of functional genes (Table 1). Duplications of *TRGV* genes have taken place in the upstream region of the J region, although remaining circumscribed in the physical area of the V region. In all examined cases (chicken, duck, shark, alligator, and opossum) except one (salmon), the *TRGC* remained unique (Table 1). 

Unique among the analyzed vertebrate species is the organization of the TRG locus in humans and in Primata (Figure 1, Table 4) where J-gene duplications also involved the *C* gene, creating a duplicated J-C cluster located downstream of the V region.

Moving from the “V-J-C-cluster” model to the structural “V-J-J-C” cassette typical of Cetartiodactyla (Ruminantia, Tylopoda, and Cetacea), the duplications involved *V*, *J*, and *C* genes physically tied together in the same chromosomal area, giving rise to “recombinational units” (seven cassettes in *Bos taurus* and six cassettes in *Ovis aries*) (Figure 2 and Table 2 and Table 3). 

The number of cassettes is reduced to three in *Camelus dromedarius* (Tylopoda). Moreover, a minimal structure V-J-C (2*V*, 3*J*, and 1*C*) with a high level of homology to the ancient Artiodactyla cassette defines the *Tursiops truncatus* (Cetacea) TRG locus.

The cassette organization is also found in Carnivora, where the number of duplicate cassettes remains high (Table 2). This representative number of eight in *Canis lupus familiaris* and six in *Felis catus* is comparable to that of ruminants. However, in contrast to the bovine and ovine TRG loci, the value of the functional/total genes ratio (Supplementary Table S1) found in Carnivora suggests that there is no correlation between the extensive duplications of the cassettes and a need for new functional genes in the adaptive immune response.

A suggestive and intriguing peculiarity of the TRG locus compared to the other TR loci is the number of *TRGC* genes and the diversity in the corresponding protein structure. In particular, the *TRGC* genes can show a different exon–intron organization that is evident not only if we compare *TRGC* genes within the TRG loci of the diverse species, but even between genes within a single TRG locus. Their diversity is mainly linked to the different number of Exon 2 copies, which can encode the connecting region. The biological significance of these repetitive exons remains unclear [83,117]. One hypothesis is the connecting-region length variation may affect processes such as signal transduction or interactions with other molecules at the cell surface [118]. In fact, the TR is capable of delivering a variety of different signals related to the different functions attributed to γδ T cells [119]. 

However, despite the differences in the TRG locus structure and in the arrangement of the genes, phylogenetic analyses show a tightly evolutionary conserved relationship of the genes encoding the variable domain in diverse species, with genes that have been preserved in all mammalian species as a result of a strong functional constraint and the emergence of new genes that have undergone substantial diversification through species-specific duplication. All these features highlight the important role of the TRG chain in the adaptive immune response.

## Figures and Tables

**Table 4:** Genomic organization and gene content of the TRG locus in Rodentia, Lagomorpha and Primata.

		TRGV Genes	TRGJ Genes	TRGC Genes	Chromosomal Localization	Miniature Locus	References
**RODENTIA**	Mouse	TRGV1 TRGV2 TRGV3 TRGV4 TRGV5 TRGV6 TRGV7	TRGJ1 TRGJ2 TRGJ3 TRGJ4	TRGC1 TRGC2 TRGC3 (P) TRGC4	One locus Chrom. 13A2	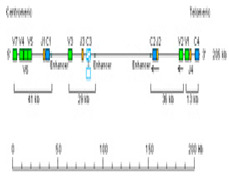 *Mus musculus*	Hayday et al., [66]; Garman et al., [105]; Traunecker et al., [106]; Pelkonen et al., [107]; Vernooij et al., [68];
**LAGOMORPHA**	Rabbit	TRGV1 (7+1O) TRGV2 (O) TRGV3 TRGV4 (O)	TRGJ1 TRGJ2	TRGC	One locus Chrom. 10	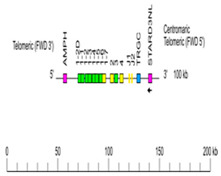 *Oryctolagus cuniculus*	Massari et al., [109];
**PRIMATA**	Rhesus monkey	TRGV1 (O) TRGV2 TRGV3 TRGV6 (P) TRGV8 TRBVA (P) TRGV9 TRGV10 (O) TRGVB (P) TRGV11 (P) TRGVC (P) TRGVD (P)	TRGJ1-1 TRGJ1-2 TRGJ2-1 TRGJ2-2 (P) TRGJ2-3	TRGC1 TRGC2	One locus Chrom. 3	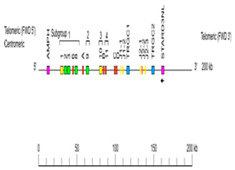 *Macaca mulatta*	Lefranc M-P., [2];
	Human	TRGV1 (O) TRGV2 TRGV3 TRGV4 TRGV5 TRGV5P (P) TRGV6 (P) TRGV7 (P) TRGV8 TRGVA (P) TRGV9 TRGV10 (O) TRGVB (P) TRGV11 (O)	TRGJP1 TRGJP TRGJ1 TRGJP2 TRGJ2	TRGC1 TRGC2	One locus Chrom. 7p14	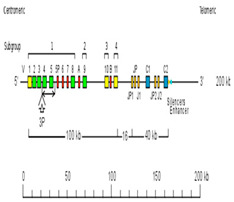 *Homo sapiens*	Lefranc, and Rabbitts, [42]; Lefranc, [43,44,45]; Lefranc and Rabbitts, [49];

Legend: P = pseudogene, O = ORF.

## Supplementary Materials

The following are available online at https://www.mdpi.com/2073-4425/11/6/624/s1, Figure S1: TRG locus representation in outgroups, Figure S2: TRG locus representation in Cetartiodactyla and Carnivora, Figure S3: TRG locus representation in Rodentia, Lagomorpha and Primata, Table S1: Ratio functional/total TRGV, TRGJ and TRGC genes.
