# Supplementary material for: Evolution of the T-Cell Receptor (TR) Loci in the Adaptive Immune Response: The Tale of the TRG Locus in Mammals
Source: Genes (Basel). 2020 Jun 5;11(6):624. doi: 10.3390/genes11060624 (PMC7349638; doi:10.3390/genes11060624)
Supplement: Supplementary file 1 [file genes-11-00624-s001.zip › Supplementary Files/Supplementary Figures (Table 1).pdf]

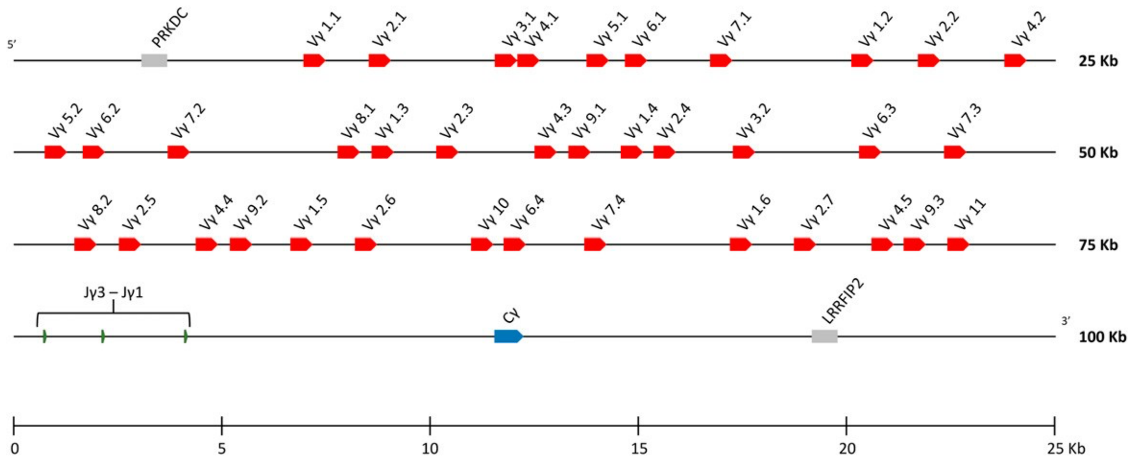

Supp. Fig. 1A
Gallus gallus (Liu et al., 2020)

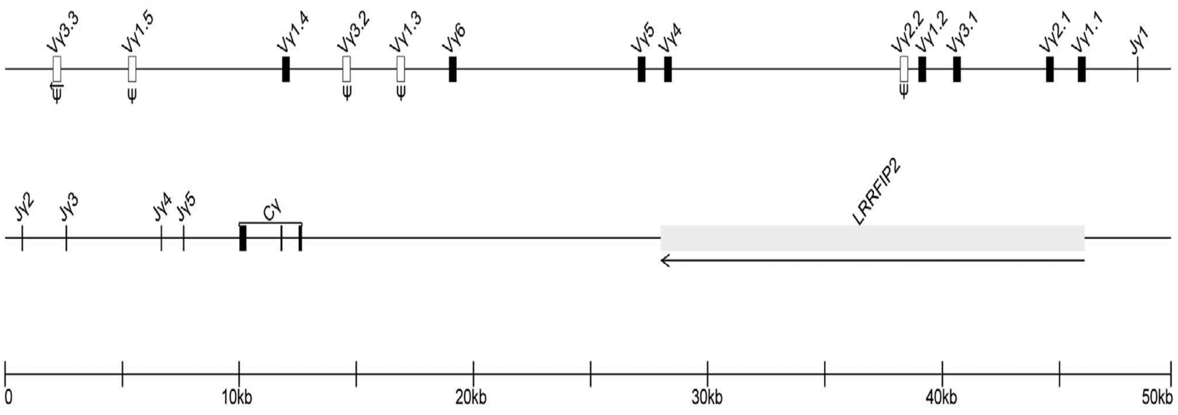

Supp. Fig. 1B
Anas platyrhynchos domesticus (Yang et al., 2017)

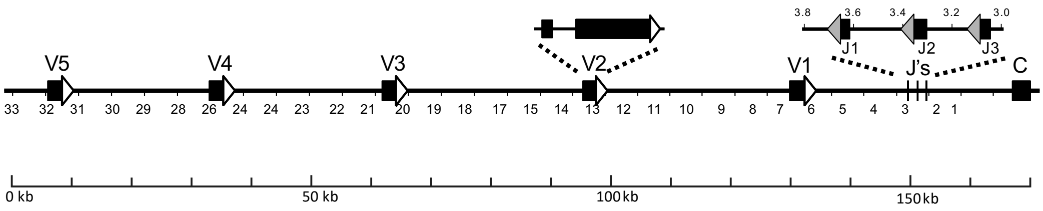

Supp. Fig. 1C
Carcharhinus plumbeus (Chen et al., 2009)

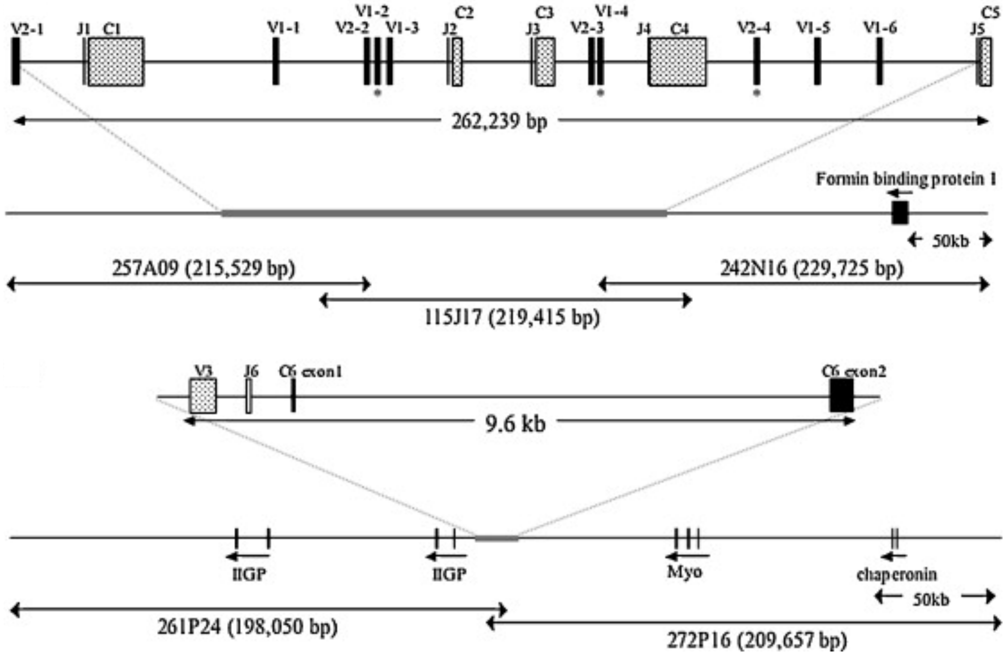

Supp. Fig. 1D
TCRγ 1 (upper) and TCRγ 2 (lower) locus *Salmo salar* (Yazawa et al., 2008)

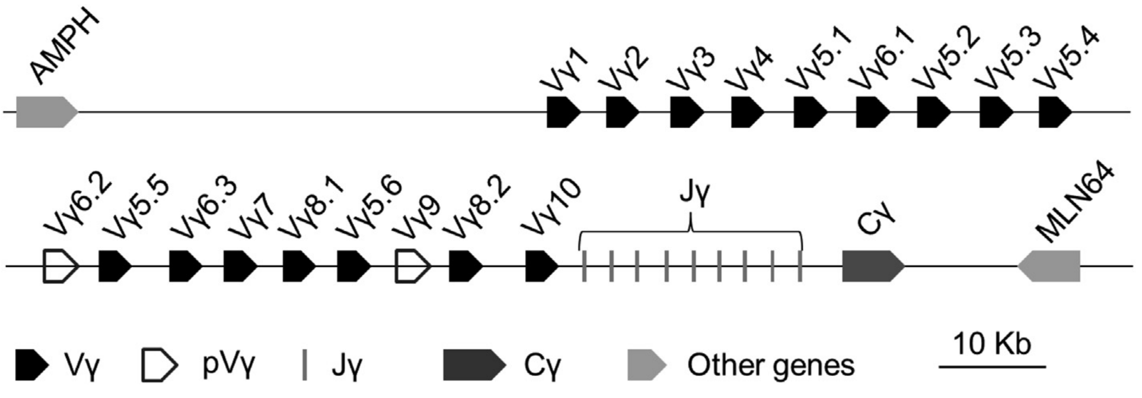

Supp. Fig. 1E
Alligator sinensis (Wang et al., 2018)

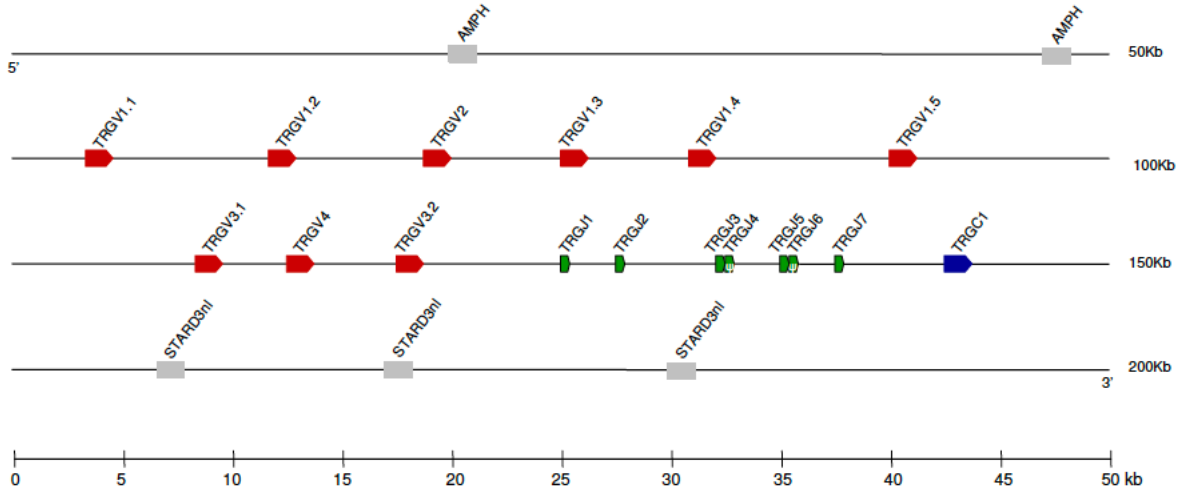

Supp. Fig. 1F
Monodelphis domestica (Parra et al., 2008)
