# Supplementary material for: Evolution of the T-Cell Receptor (TR) Loci in the Adaptive Immune Response: The Tale of the TRG Locus in Mammals
Source: Genes (Basel). 2020 Jun 5;11(6):624. doi: 10.3390/genes11060624 (PMC7349638; doi:10.3390/genes11060624)
Supplement: Supplementary file 1 [file genes-11-00624-s001.zip › Supplementary Files/Supplementary Figures (Table 2A).pdf]

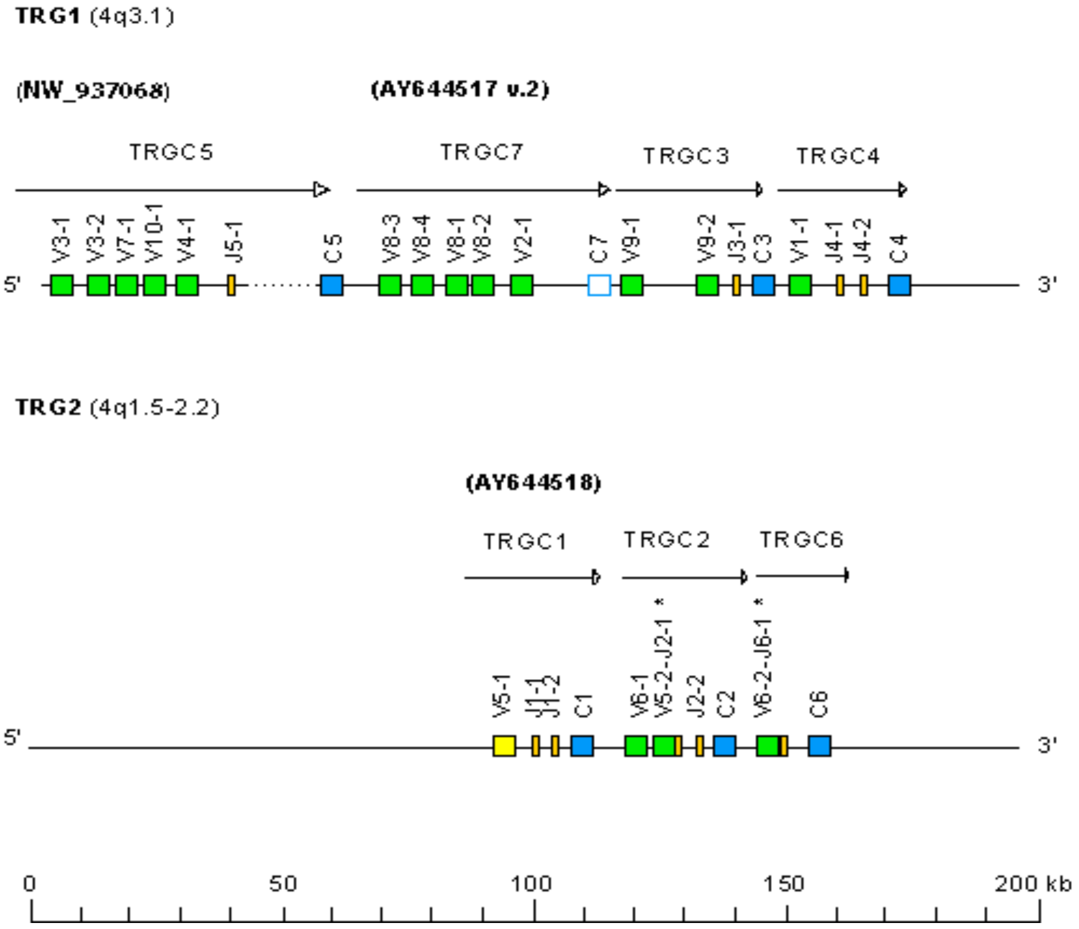

Supp. Fig. 2A
Bos taurus (with permission of IMGT® http://www.imgt.org)

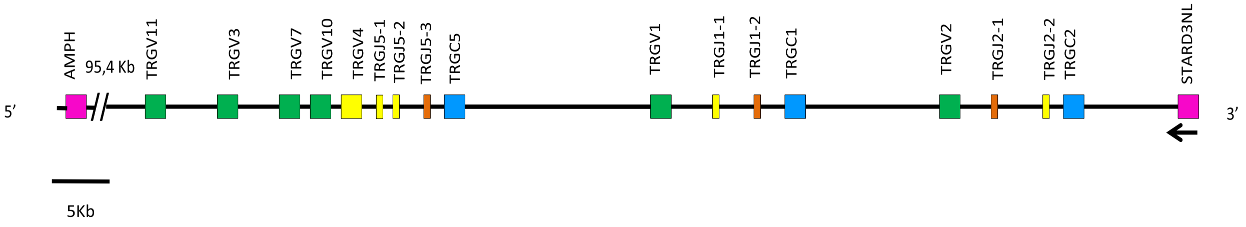

Supp. Fig. 2B
Camelus dromedarius (Antonacci et al., 2020)

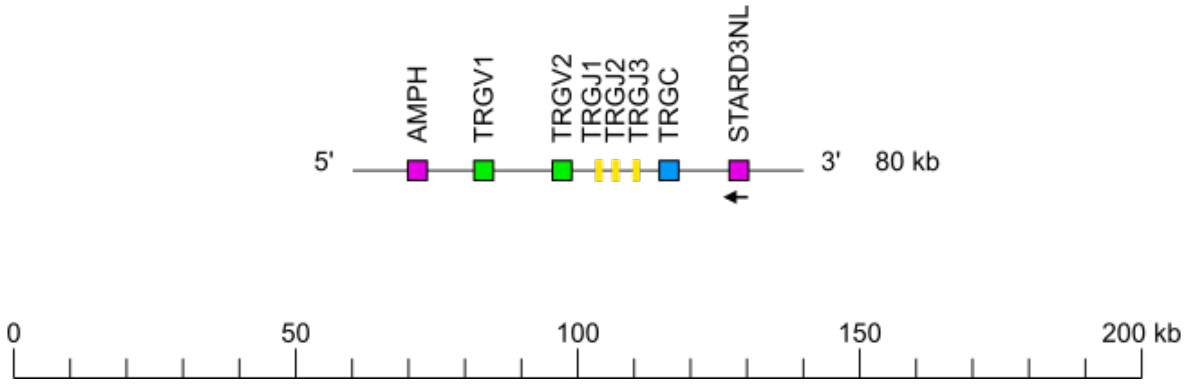

Supp. Fig. 2C
Tursiops truncatus (with permission of IMGT® http://www.imgt.org)

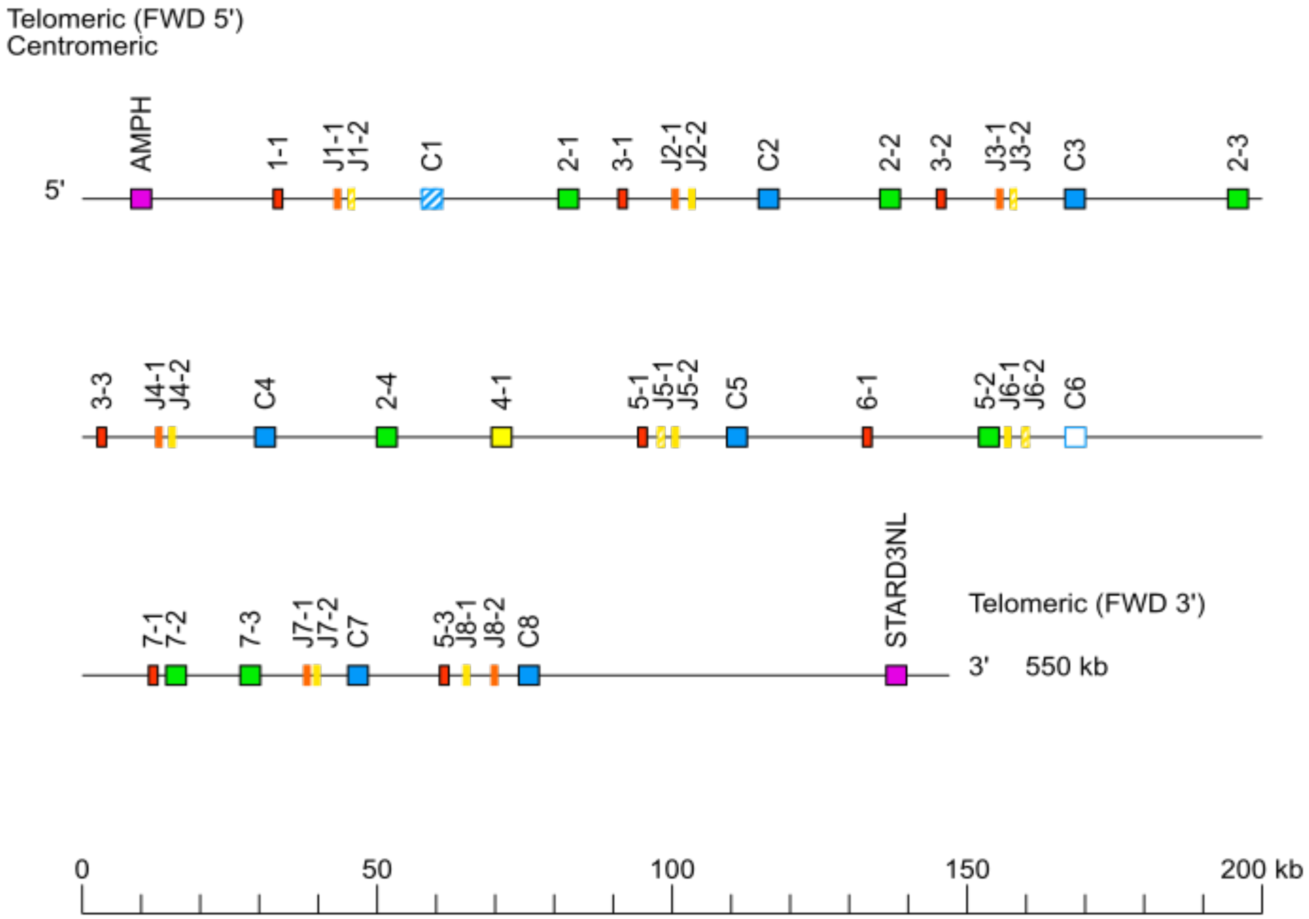

Supp. Fig. 2D
Canis lupus familiaris (with permission of IMGT® http://www.imgt.org)

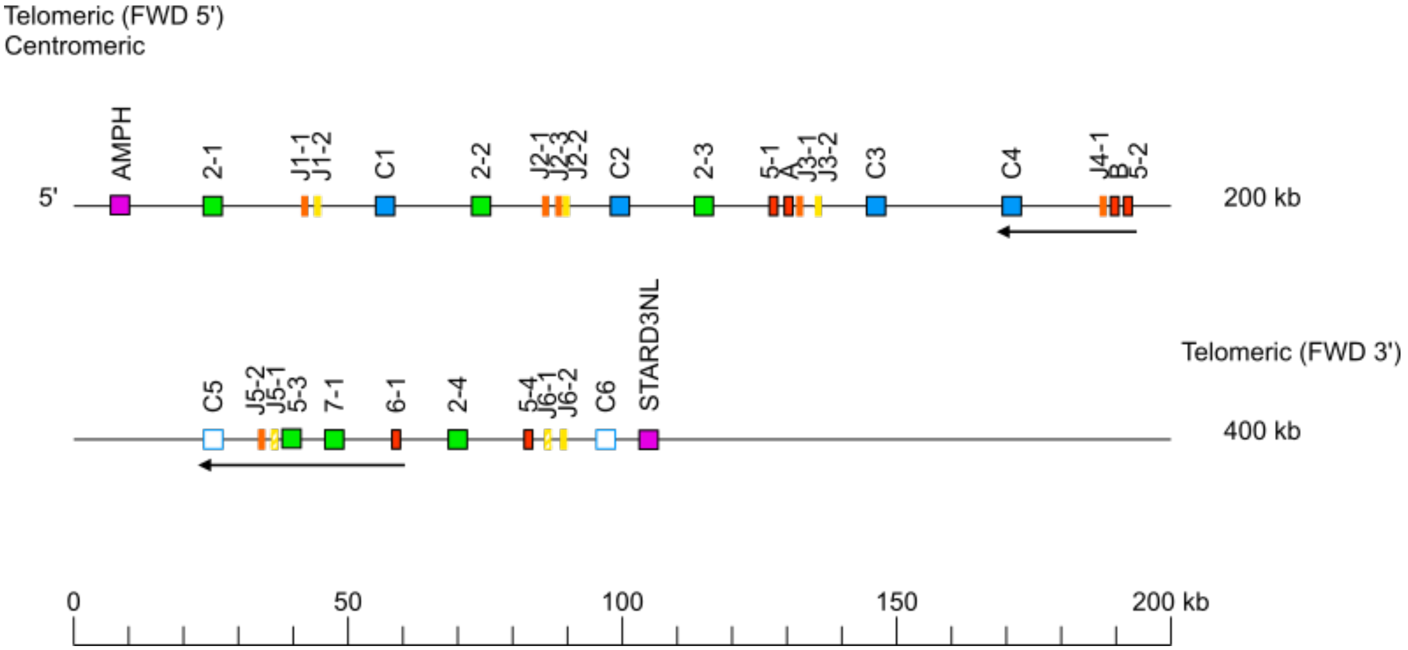

Supp. Fig. 2E
Felis catus (with permission of IMGT® http://www.imgt.org)
