# Supplementary material for: Evolution of the T-Cell Receptor (TR) Loci in the Adaptive Immune Response: The Tale of the TRG Locus in Mammals
Source: Genes (Basel). 2020 Jun 5;11(6):624. doi: 10.3390/genes11060624 (PMC7349638; doi:10.3390/genes11060624)
Supplement: Supplementary file 1 [file genes-11-00624-s001.zip › Supplementary Files/Supplementary Table 1.pdf]

Supplementary Table 1. Ratio functional/total TRGV, TRGJ and TRGC genes

Gene	*Functionality*	*Ovis aries*	*Bos taurus*	*Camelus dromedarius*	*Tursiops truncatus*	*Canis lupus familiaris*	*Felis catus*
**TRGV**	F	11	16	6	2	7	6
	ORF			1		1	
	P	2	1			8	6
**TRGJ**	F	10	10	4	3	10	6
	ORF						
	P	3		3		6	6
**TRGC**	F	6	6	3	1	6	4
	ORF					1	
	P		1			1	2
**Total**		32	34	17	6	40	30
**Ratio Functional /total**		27/32<br>0,77	32/34<br>0,94	13/17<br>0,76	6/6<br>1	23/40<br>0,57	16/30<br>0,53
