# Supplementary material for: Evolution of the T-Cell Receptor (TR) Loci in the Adaptive Immune Response: The Tale of the TRG Locus in Mammals
Source: Genes (Basel). 2020 Jun 5;11(6):624. doi: 10.3390/genes11060624 (PMC7349638; doi:10.3390/genes11060624)
Supplement: Supplementary file 1 [file genes-11-00624-s001.zip › Supplementary Files/Supplementary Figures (Table 3).pdf]

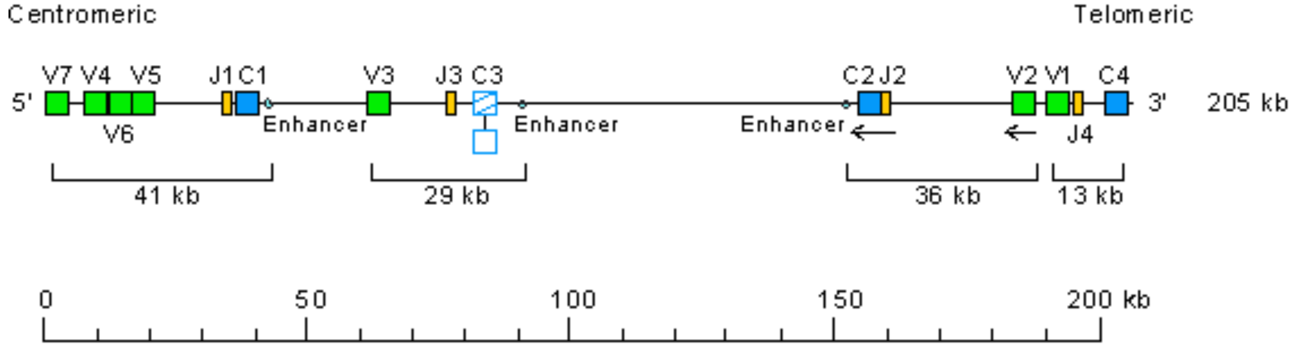

Supp. Fig. 3A
Mus musculus (with permission of IMGT® http://www.imgt.org)

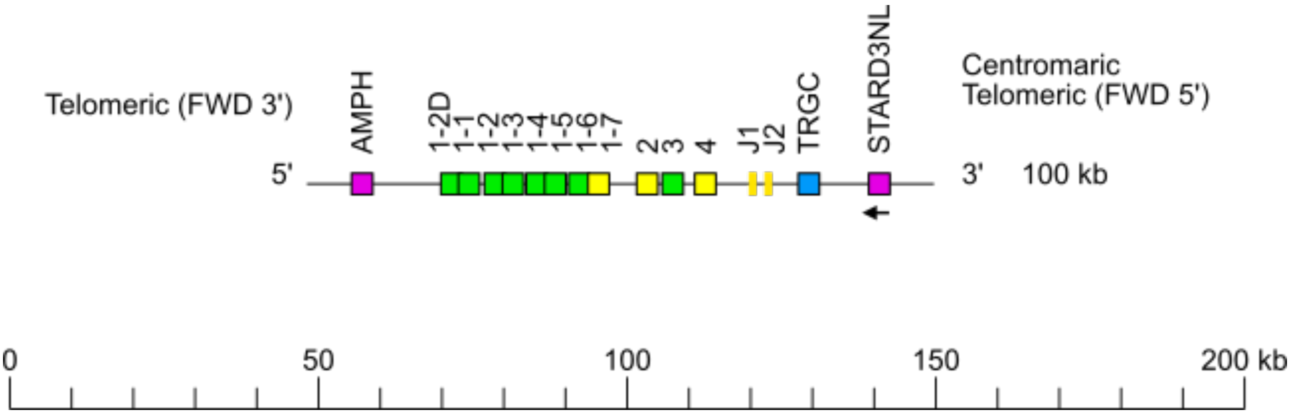

Supp. Fig. 3B
Oryctolagus cuniculus (with permission of IMGT® http://www.imgt.org)

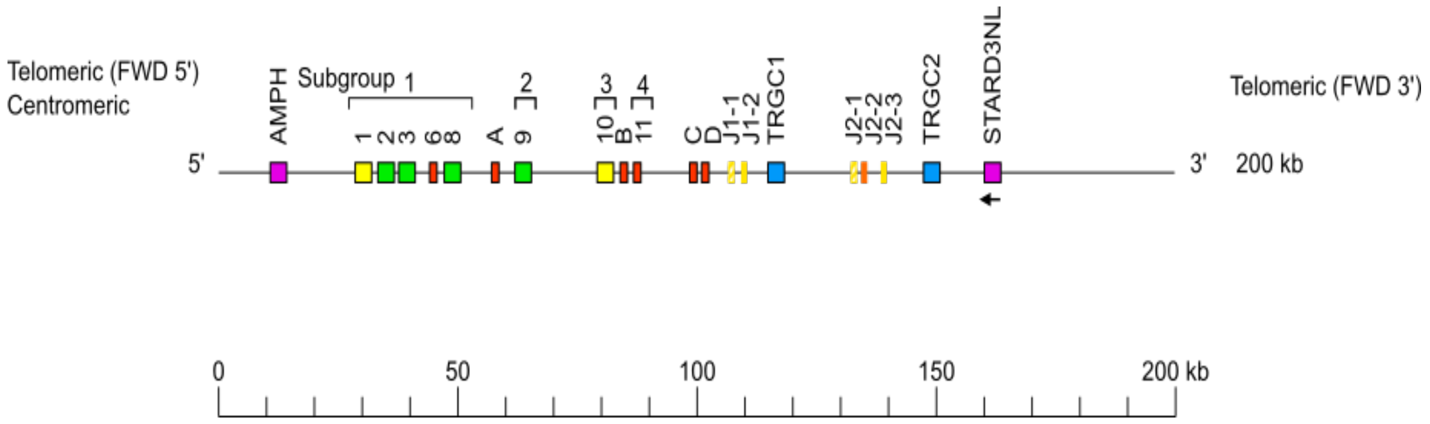

Supp. Fig. 3C
Macaca mulatta (with permission of IMGT® http://www.imgt.org)
